# Asprosin‐FABP5 Interaction Modulates Mitochondrial Fatty Acid Oxidation through PPARα Contributing to MASLD Development

**DOI:** 10.1002/advs.202415846

**Published:** 2025-04-15

**Authors:** Yuan‐Yuan Yu, Min Feng, Yi Chen, Hong‐Lin Jia, Qi Zhang, Ming Tong, Yan‐Xi Li, Yu Zhao, Xin‐Xin Liu, Shi‐Feng Cao, Zheng‐kai Wang, Hou‐wei Li, Xue Liu, Yan Zhang

**Affiliations:** ^1^ Department of Pharmacology SKLFZCD (State Key Laboratory ‐Province Key Laboratories of Biomedicine‐Pharmaceutics of China Key Laboratory of Cardiovascular Research Ministry of Education) College of Pharmacy Harbin Medical University Harbin 150081 China; ^2^ Department of Pathophysiology (Province Key Laboratory of Medicine‐Food Homologous Resources and Prevention and Treatment of Metabolic Diseases) Basic Medical College Qiqihar Medical University Qiqihar 161000 China; ^3^ Department of Cardiology at the Second Affiliated Hospital of Harbin Medical University State Key Laboratory of Frigid Zone Cardiovascular Diseases Harbin 150086 China; ^4^ Department of Pharmacology State Key Laboratory of Frigid Zone Cardiovascular Diseases (SKLFZCD) Department of Pharmacy of The Second Affiliated Hospital Harbin Medical University Harbin 150081 China

**Keywords:** asprosin, FABP5, fenofibrate, hepatic steatosis, insulin resistance, mitochondrial fatty acid β‐oxidation, PPARα

## Abstract

Alterations in liver metabolism play a pivotal role in the development and progression of metabolic dysfunction‐associated steatotic liver disease (MASLD). Asprosin is reported to be released from white adipose tissue during fasting and targets the liver. However, the role of asprosin, especially from organs other than adipose tissue, in MASLD remains poorly understood. These findings demonstrate that plasma asprosin levels are significantly elevated in MASLD patients and animal models. Additionally, asprosin expression increased in the liver of MASLD mice. Hepatocyte‐specific overexpression of asprosin impairs mitochondrial fatty acid β‐oxidation (FAO), whereas its knockdown not only enhances FAO in mice but also compensates for fenofibrate's limitations in MASLD treatment. Mechanistic investigations reveal that the interaction of asprosin with FABP5 facilitates its abnormal nuclear localization, and asprosin directly bound to and inhibites peroxisome proliferator‐activated receptor elements (PPREs), which negatively regulated PPARα transcriptional activity, and disrupts hepatic FAO pathways. GalNAc‐siRNAs targeting hepatic FABP5 ameliorate hepatic steatosis. These findings reveal that the secretory adipose factor asprosin is expected to act as a biological marker for early clinical diagnosis and prognostic evaluation of MASLD. Moreover, targeting hepatic asprosin gene inhibition and GalNAc‐siRNAs to inhibit hepatic FABP5 both offer potential therapeutic benefits in the treatment of MASLD.

## Graphical abstract

Aided by FABP5, abnormally elevated asprosin in hepatocytes enters the nucleus, targets and inhibits PPARα binding to the CPT1A promoter, thereby suppressing FAO. Circulating asprosin exacerbates insulin resistance, collectively driving MASLD progression. Targeted inhibition of the hepatic asprosin gene and GalNA siRNAs against hepatic FABP5 may offer therapeutic advantages for the treatment of MASLD.

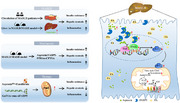

## 1 Introduction

Metabolic dysfunction‐associated steatotic liver disease (MASLD) currently affects more than 30% of the adult population worldwide, making it one of the leading causes of liver disease worldwide.^[^ ^1^, ^2^ ^]^ MASLD‐accompanied hepatic steatosis is usually reversible, but it can progress from simple steatosis to metabolic dysfunction‐associated steatohepatitis (MASH), which can progress to cirrhosis or hepatocellular carcinoma (HCC).^[^ ^3^ ^]^ As a metabolic‐related disease, MASLD prevalence among overweight individuals ranges from 22.5% to 44.0% and reaches as high as 90% among obese individuals.^[^ ^4^, ^5^, ^6^ ^]^ Although MASLD is a serious risk to human health and has a substantial economic cost, current management primarily focuses on lifestyle interventions, including low‐calorie diet, exercise, and weight reduction, and early treatment of cardiometabolic risk factors, with no specific approved drugs for MASLD or MASH.^[^ ^7^ ^]^ There is no standardized medication for MASLD mainly due to unclear pathogenesis and undefined effective therapeutic targets, therefore, it is of great clinical significance to conduct in‐depth research into the pathogenesis and identify effective intervention targets for MASLD.

Obesity is the major risk factor for MASLD. Adipocyte dysfunction leads to dysregulation of adipokines, affecting glucose and lipid stability, insulin resistance, and inflammatory responses, leading to a variety of metabolic diseases.^[^ ^8^ ^]^ Adipose tissue can act as an endocrine organ to secrete adipose‐secreted proteins (adipokines), which regulate glucose metabolism and lipid metabolism.^[^ ^9^ ^]^ Persistent obesity and chronic inflammation lead to adipose tissue dysfunction, causing changes in adipokine secretion. Adipokines are involved in the regulation of liver function, and dysregulation of adipokines contributes to the development of MASLD.^[^ ^10^ ^]^

Romere et al^[^ ^11^ ^]^ first identified a gluconeogenic adipokine in patients with neonatal progeroid syndrome (NPS), called asprosin, which is a C‐terminal cleavage product of protofibrillin‐1 (FBN1) in the presence of proteases.^[^ ^12^ ^]^ There is growing evidence that as a novel adipokine, asprosin may affect appetite, causing insulin resistance, and damage to pancreatic β‐cells. By promoting endoplasmic reticulum stress, it leads to insulin resistance in skeletal muscle. In addition, the elevated circulating asprosin level is closely associated with clinical relevant parameters of metabolic disorders such as type 2 diabetes, obesity, MASLD.^[^ ^13^, ^14^ ^]^ However, the precise role of asprosin in the progression of MASLD is still not well understood.

## 2 Results

### 2.1 Asprosin Level Increases in MASLD Patients and Experiment Animals

**Figure 1:**
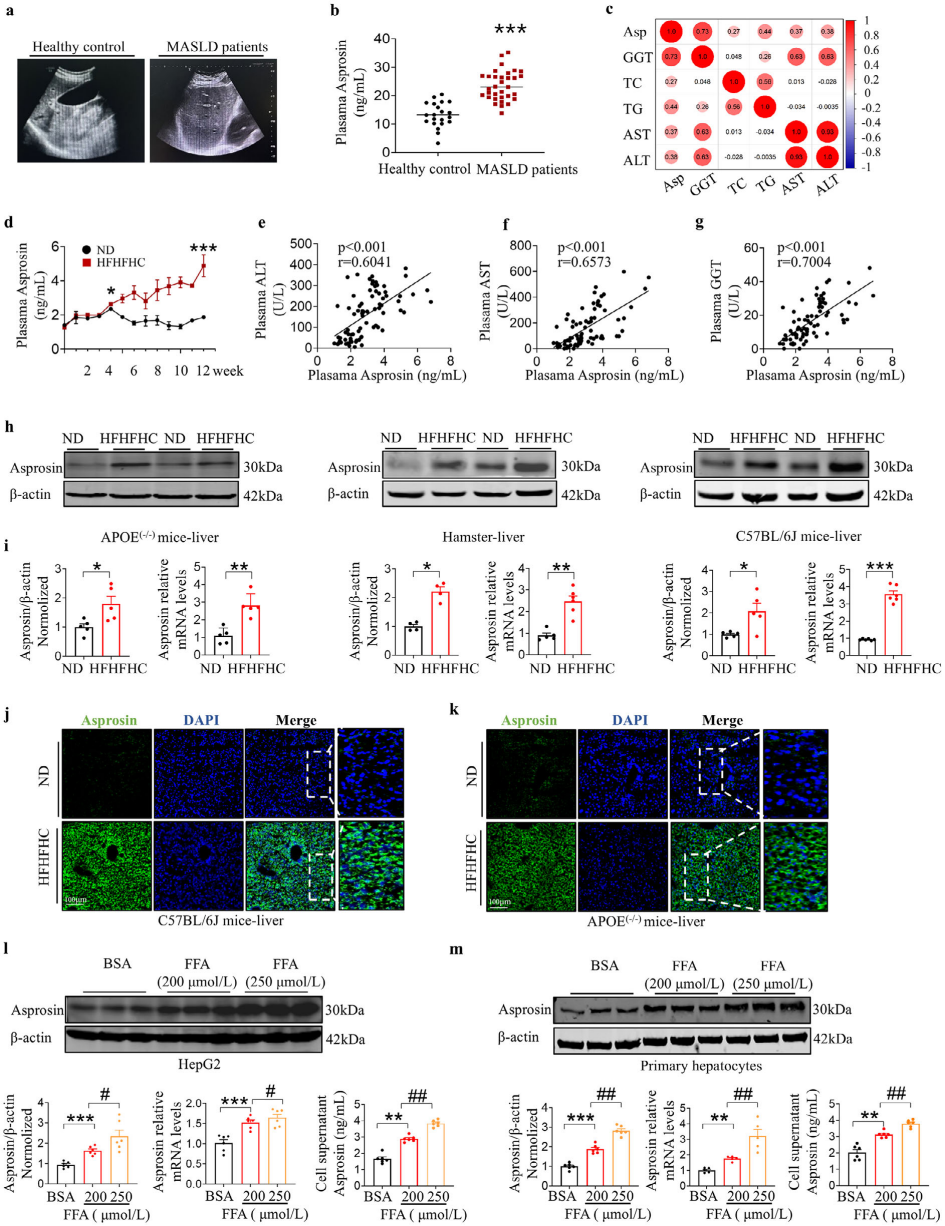
Asprosin levels increases in MASLD patients and animals. a) Ultrasound images of the liver in healthy controls and MASLD patients. b) Detection of plasma asprosin levels in healthy controls and MASLD patients. *n* = 21, 33 in each group. c) Heatmap for correlation analysis of asprosin and GGT, TC, TG, ALT, AST levels in plasma of healthy controls and MASLD patients. *n* = 52 in each group. d) Weekly measurements of serum asprosin levels in mice fed a high‐fat, high‐cholesterol, high‐fructose (HFHFHC) diet over a period of 12 weeks. *n* = 6 in each group. e–g) Analysis of the correlation between asprosin levels and liver enzymes (ALT, AST and GGT) in mouse serum under high‐fat, high‐cholesterol, and high‐fructose diet model. *n* = 7, 8 in each group. h,i) Levels of hepatic asprosin protein and mRNA in different MASLD models: APOE^(‐/‐)^ mice on a HFHFHC diet for 21 weeks, hamsters on HFHFHC diet for 4 weeks, and C57BL/6J mice on HFHFHC diet for 12 weeks. *n* = 4, 5 in each group. j,k) Immunofluorescence detection of asprosin in the livers of various groups of mice. *n* = 4 in each group. (l‐m) Different concentrations of FFA induce changes in asprosin protein and mRNA in HepG2 cells and primary hepatocytes; Changes in asprosin levels in supernatants of HepG2 cells and primary hepatocytes. HepG2 cells and primary hepatocytes were stimulated with free fatty acids (FFA, 200 µM, 250 µM) for 24 h. *n* = 6 in each group. Scale bar: 100 µm. HFHFHC, high‐fat, high‐cholesterol, high‐fructose; ALT, aspartate aminotransferase; AST, alanine aminotransferase; GGT, gamma‐glutamyl transferase; ND, normal diet; FFA, free fatty acids. Statistical analysis was performed with one‐way ANOVA. *p < 0.05 versus ND, **p < 0.01 versus ND, ***p < 0.001 versus ND, **p < 0.01 versus BSA, ***p < 0.001 versus BSA, #*p* < 0.05 versus FFA (200 µmol/L), ##*p*,< 0.01 versus FFA (200 µmol/L).

To investigate the clinical association between asprosin and MASLD, we measured plasma asprosin levels in MASLD patients and individuals with normal liver function, and found that plasma asprosin levels in MASLD patients were significantly elevated compared to healthy controls (**Figure** **1**a,b). A positive correlation was also observed between asprosin levels and liver injury markers gamma‐glutamyltransferase (GGT), aspartate transaminase (AST), alanine amino transferase (ALT) as well as triglyceride (TG), and total cholesterol (TC) (Figure 1c; Figure S1a, Supporting Information). In this study, male C57BL/6J mice were continuously fed a high‐fat, high‐cholesterol, and high‐fructose (HFHFHC) diet for 12 weeks to induce liver lesions that mimic the clinical characteristics of MASLD.^[^ ^15^ ^]^ Plasma asprosin levels were initially measured weekly in C57BL/6J mice (Figure 1d). After 4 weeks on the HFHFHC diet, plasma asprosin levels in MASLD mice significantly increased compared to those in the normal diet group. Moreover, plasma asprosin levels in mice positively correlated with ALT, AST, and GGT levels (Figure 1e–g, Figure S1b, Supporting Information). In addition, we established models of HFHFHC diet‐induced hepatic steatosis in APOE^(‐/‐)^ mice, hamsters, and C57BL/6J mice. As shown in Figure 1h–k, the HFHFHC diet induced a significant increase in asprosin protein and mRNA expression in the livers of APOE^(‐/‐)^ mice, hamsters, and C57BL/6J mice. HepG2 cells were chosen for in vitro experiments due to their human origin, which closely resembles human liver tissue. Additionally, free fatty acids (FFA) can induce lipid deposition in HepG2 cells, making them more suitable for studying the pathogenesis of MASLD.^[^ ^16^ ^]^ FFA stimulation significantly increased asprosin protein and mRNA levels in HepG2 cells, and the levels of asprosin in the supernatants of HepG2 cells were also increased by FFA induction (Figure 1k–l). The same results were obtained in primary hepatocytes (Figure 1m ).

### 2.2 Hepatic Asprosin Deficiency Alleviates Hepatic Steatosis

**Figure 2:**
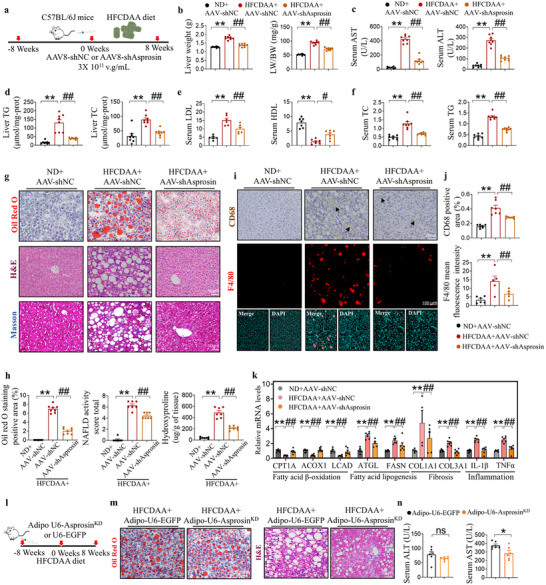
Hepatic asprosin deficiency alleviates hepatic steatosis. a) Schematic illustration of the experimental design employed to assess the impact of hepatic asprosin deficiency on hepatic steatosis. b) Liver weight, and LW/BW of C57BL/6J mice from different groups. *n* = 8 in each group. c) AAV‐shAsprosin reduced the elevated serum transaminase level induced by HFCDAA feeding in C57BL/6J mice. *n* = 6–8 in each group. d) The total cholesterol and triglyceride content of the livers. *n* = 8 in each group. e,f) The serum TG, TC, LDL, HDL levels. *n* = 8 in each group. g,h) Representative histological sections stained with Oil Red O, hematoxylin and eosin (H&E), and Masson's trichrome. Quantitative analyses of Oil Red O positive areas, non‐alcoholic fatty liver disease activity score (NAS), and detection of liver hydroxyproline content are shown. *n* = 6–8 in each group. i,j) Immunohistochemical staining for CD68 and immunostaining staining for F4/80. Quantitative of CD68 positive areas and mean fluorescence intensity of F4/80 staining are shown. *n* = 5–7 in each group. k) AAV‐shAsprosin changed the expression of genes involved in lipid metabolism. *n* = 6–8 in each group. l) Schematic illustration of the experimental design employed to assess the impact of adipose asprosin deficiency on hepatic steatosis. m) Representative histological sections stained with Oil Red O, hematoxylin, and eosin (H&E). *n* = 6 in each group. n) The serum ALT, AST levels. *n* = 6 in each group. LW/BW, liver weight/body weight, TC, total cholesterol; TG, triglyceride; ALT, aspartate aminotransferase; AST, alanine aminotransferase; HDL‐C, high‐density lipoprotein cholesterol; LDL‐C, low‐density lipoprotein cholesterol; ND, normal diet; HFCDAA, high fat, methionine choline deficiency diet. Scale bar for Oil Red O, H&E, Masson's trichrome staining, and CD68 immunohistochemical: 50 µm, for F4/80 immunostaining: 100 µm. Statistical analysis was performed with one‐way ANOVA. **p < 0.01 versus ND+AAV‐shNC. #*p* < 0.05, ##*p* < 0.01 versus HFCDAA+AAV‐shNC.

To conduct an in‐depth study on the effect of asprosin on MASLD progression, mice were treated with recombinant AAV8 containing asprosin shRNA to knock down asprosin (asprosin^KD^) in the liver. Western blot analysis confirmed the knockdown efficiency (Figure S2a, Supporting Information). A MASH mouse model was induced in C57BL/6 mice by feeding them an HFCDAA diet for 8 weeks.^[^ ^17^ ^]^ Before starting the diet, mice were administered either rAAV‐shAsprosin or a non‐targeting control (rAAV‐shNC) via tail vein injection (**Figure** **2**a). As depicted in Figure S2b (Supporting Information), unlike the normal‐sized, reddish‐brown livers of ND‐fed C57BL/6 mice, those of HFCDAA‐fed C57BL/6 mice became swollen and yellow; AAV‐shAsprosin treatment partially restored the liver color and size (Figure S2b, Supporting Information). Liver weights and liver/body weights of C57BL/6 mice with the treatment are shown in Figure 2b. Transaminase levels were reduced in AAV‐shAsprosin‐treated mice, indicating alleviated liver damage (Figure 2c; Figure S2d, Supporting Information). Furthermore, serum TG, TC, and LDL‐C were reduced in AAV‐shAsprosin mice compared to HFCDAA mice, and the abnormal reduction of HDL‐C levels induced by the HFCDAA diet was reversed (Figure 2e,f), and circulating asprosin levels were significantly reduced in AAV‐shAsprosin mice (Figure S2e, Supporting Information). The NAFLD Activity Score (NAS), a semi‐quantitative histological scoring system for MASLD progression, indicated significantly higher scores in the HFCDAA‐fed group. Asprosin^KD^ improved HFCDAA‐induced steatosis, inflammation, and hepatocyte ballooning (Figure 2g,h, Supporting Information). Liver TC, TG levels were consistent with this observation (Figure 2d), and inflammatory infiltration and fibrosis progression were reduced in asprosin^KD^ mice with the HFCDAA diet (Figure 2i,j, Supporting Information). Asprosin deficiency significantly increased the expression of genes involved in fatty acid β‐oxidation, while inhibiting those associated with fatty acid lipogenesis, proinflammation, and profibrogeneis, including ATGL, FASN, COL1A1, COL3A1, TNFα, and IL‐1β. Collectively, these data suggest that hepatic asprosin^KD^ effectively alleviates MASH progression (Figure 2k).

Prior studies have indicated that white adipose tissue is the primary source of asprosin. We developed an adipose tissue‐specific knockdown model by injecting adeno‐associated virus into mice, which were also fed an HFCDAA diet for 8 weeks (Figure 2l). Western blot analysis confirmed the knockdown efficiency (Figure S2e, Supporting Information). Adipo‐asprosin^KD^ did not reverse HFCDAA diet‐induced hepatic lipid accumulation in C57BL/6J mouse livers as evidenced by Oil Red O. Similar results were observed with HE staining, which showed persistent fatty vacuoles and inflammatory infiltrates in the livers of adipo‐asprosin^KD^ mice (Figure 2m). Serum ALT and AST levels in adipo‐asprosin^KD^ mice remained elevated and had no significant effect on lipid profiles (Figure 2n, Figure S2f, Supporting Information). These findings suggest that adipose‐derived asprosin may be insufficient to exacerbate MASLD progression, whereas liver‐derived asprosin plays a more critical role in promoting MASLD pathology.

### 2.3 Asprosin Induces Lipid Accumulation in Hepatocytes

**Figure 3:**
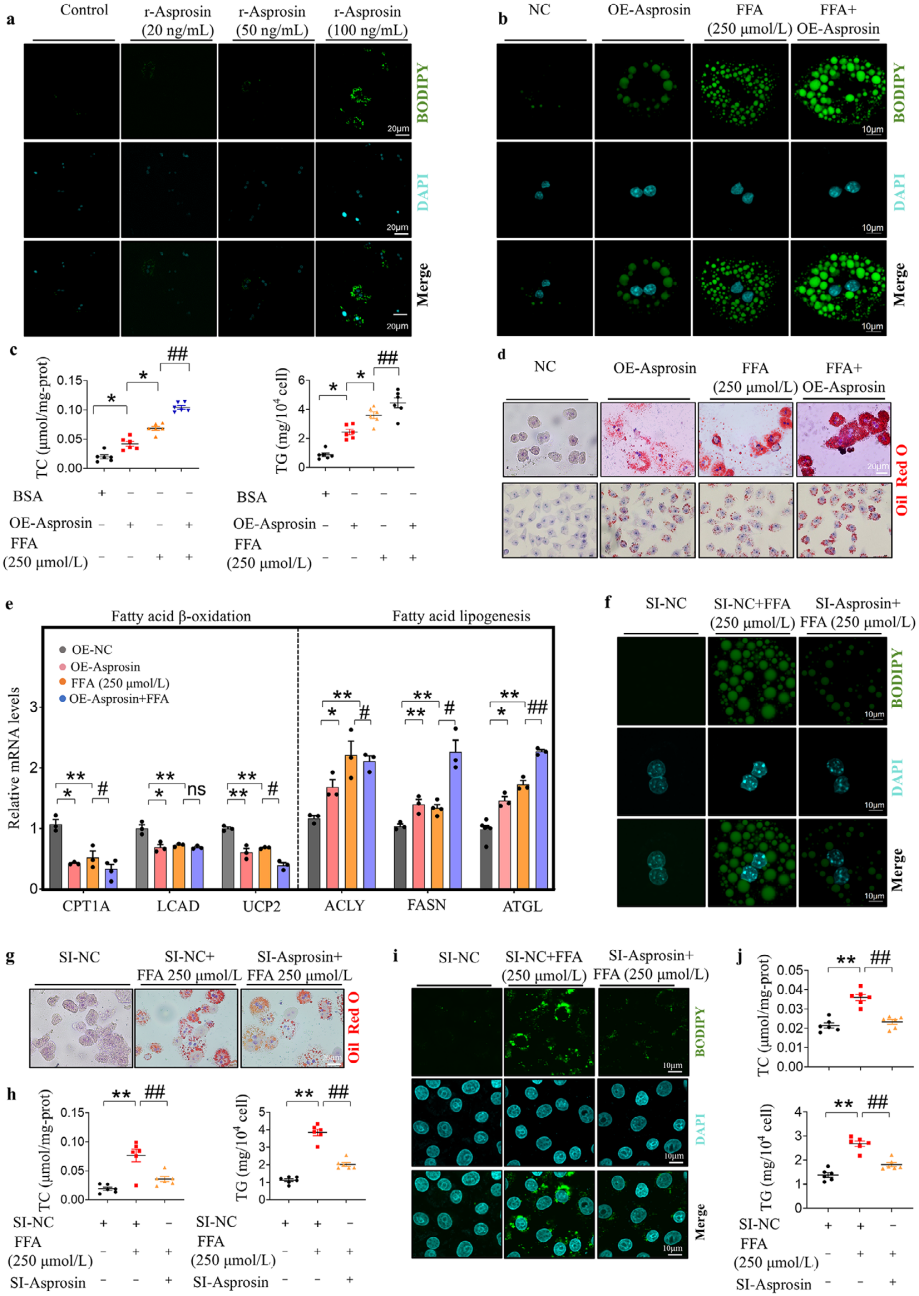
Asprosin induces lipid accumulation in hepatocytes. a) Primary hepatocytes were treated with different concentrations of asposin recombinant protein (r‐Asprosin, 20, 50, 100 ng mL^−1^) for 24 h. *n* = 6 in each group. b,d) Representative images of Oil Red O and BODIPY staining in primary hepatocytes and HepG2 cells. Cells were transfected with an asprosin overexpression plasmid for 24 h, followed by stimulation with 250 µM free fatty acids (FFA) for another 24 h. *n* = 5, 6 in each group. c) Detection of TC, TG content in primary hepatocytes. *n* = 6 in each group. e) Quantitative PCR was performed to determine the hepatic mRNA levels of genes related to fatty acid metabolism. *n* = 5, 6 in each group. f,g) Effects of asprosin knockdown on lipid accumulation in primary hepatocytes induced by 250 µM FFA, evidenced by reduced Oil Red O and BODIPY staining. *n* = 6 in each group. h) Detection of TC, TG content in primary hepatocytes. *n* = 6 in each group. i) BODIPY staining in HepG2 cells. j) Detection of TC, TG content in HepG2 cells. *n* = 6 in each group. Scale bar for Oil red O staining: 20 µm or 50 µm, for BODIPY staining: 10 µm or 20 µm. Statistical analysis was performed with one‐way ANOVA. *p < 0.05 versus NC, **p < 0.01 versus NC, #*p* < 0.05 versus FFA (250 µmol/L), ##*p* < 0.01 versus FFA (250 µmol/L).

To explore the effects of asprosin on lipid accumulation in hepatocytes, we treated primary hepatocytes with recombinant asprosin protein, and found that neither 20 nor 50 ng mL^−1^ of recombinant asprosin protein induced lipid accumulation, while 100 ng mL^−1^ of exogenous asprosin caused a slight increase in hepatocellular lipid content (**Figure** **3**a). Asprosin overexpression was also established using plasmid in primary hepatocytes. The efficiency of asprosin overexpression is shown in (Figure S3a, Supporting Information). Overexpression asprosin not only induced lipid accumulation in primary hepatocytes but also aggravated FFA‐induced lipid accumulation demonstrated with Oil red O staining and BODIPY staining. Additionally, overexpression asprosin elevated TC and TG levels in these cells (Figure 3b–d, Figure S3b,c, Supporting Information). We further analyzed the effect of over expression asprosin on β‐oxidation related gene expression in hepatocytes. Overall, asprosin inhibited the expression of genes, such as CPT1A, UCP1, LCAD, while increasing the expression of fatty acid lipogenesis related genes, including ACLY, FASN, and ATGL (Figure 3e). We also performed asprosin knockdown experiments in primary hepatocytes, and the knockdown efficiency is shown in Figure S3d,e (Supporting Information). Knockdown of asprosin significantly inhibited FFA‐induced lipid accumulation (250 µM) (Figure 3f–h, Figure S3f,g, Supporting Information). Similar results were observed in HepG2 cells (Figure 3i,j, Figure S3h,i, Supporting Information).

### 2.4 Asprosin Exacerbates HFHFHC Diet Induced Hepatic Steatosis, Hyperlipidaemia and Increases Insulin Resistance

**Figure 4:**
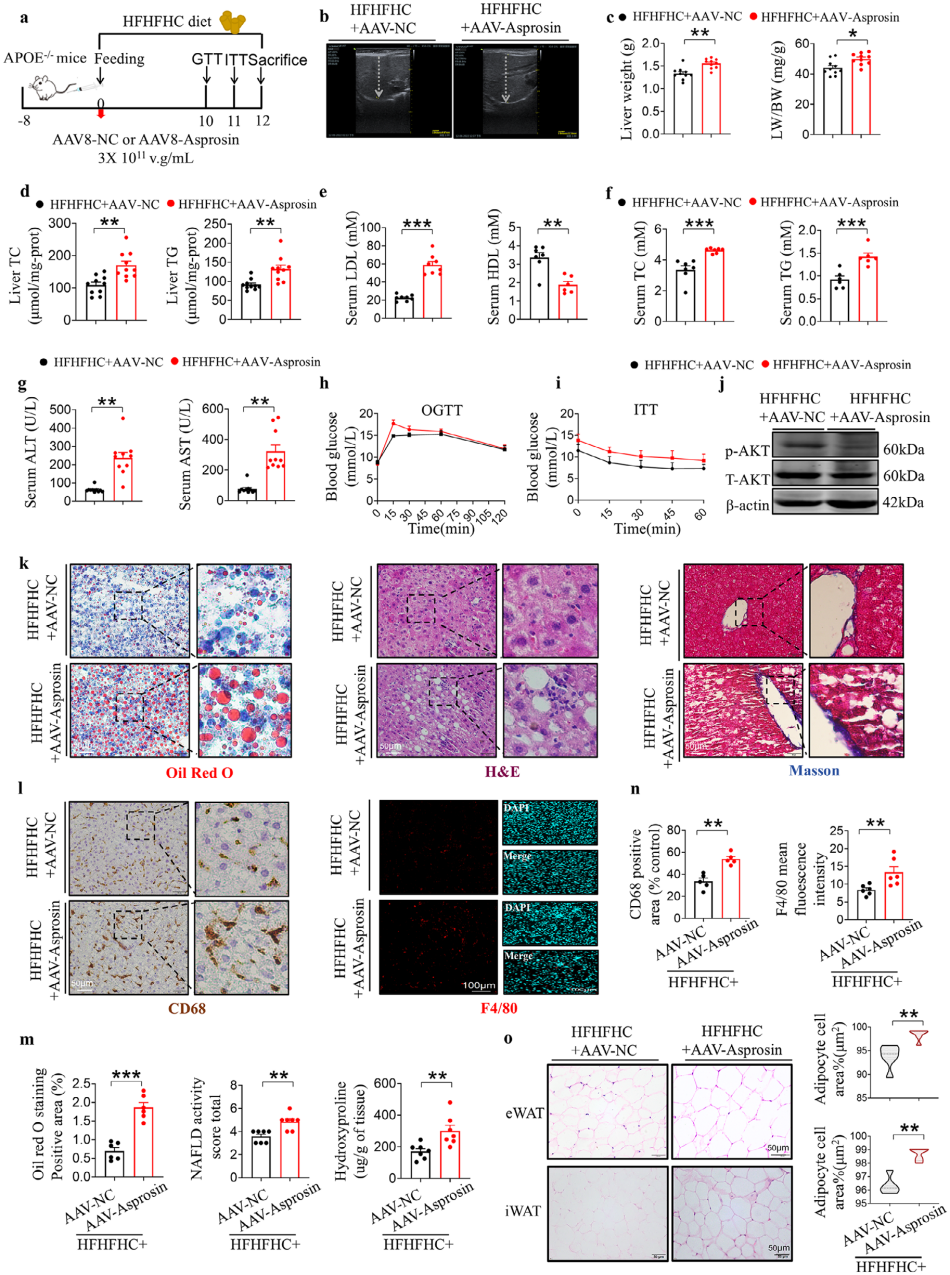
Asprosin exacerbates HFHFHC diet induced hepatic steatosis, hyperlipidemia, and increases insulin resistance. a) Schematic illustration of the experiment design. b) Representative Doppler ultrasound images showing the oblique diameter of the right lobe of the liver. *n* = 11 in each group. c) Liver weight, and LW/BW of APOE^(^^−/−)^ mice from different groups. *n* = 9 in each group. d–g) The serum LDL, HDL, TG, TC, AST, ALT levels and liver TC, TG levels. *n* = 7–10 in each group. h,i) Results from Oral Glucose Tolerance Tests (OGTT) and Insulin Tolerance Tests (ITT) performed at weeks 10 and 11, respectively, to evaluate glucose metabolism and insulin sensitivity. *n* = 6 in each group. j) Western blot analysis depicting levels of phosphorylated AKT (p‐AKT) and total AKT in the livers of insulin‐stimulated APOE^(‐/‐)^ mice on the HFHFHC diet. *n* = 6 in each group. k–l) Histological examination of liver biopsies stained with Oil Red O, hematoxylin and eosin (H&E), and Masson's trichrome, alongside immunohistochemical staining for CD68 and immunostaining for F4/80. *n* = 6, 7 in each group. m,n) The statistics of Oil Red O‐positive areas, non‐alcoholic fatty liver disease activity score (NAS), quantitative of F4/80 mean fluorescence intensity of liver, and CD68 positive areas in liver sections are shown. The detection of liver hydroxyproline of liver content is shown. *n* = 6, 7 in each group. o) H&E staining images of epididymal adipose and subcutaneous white fat tissue and quantification of epididymal adipose tissue area. *n* = 5, 6 in each group. Scale bar for Oil Red O, H&E, Masson's trichrome, CD68 immunohistochemical staining: 50 µm, for F4/80 immunostaining: 100 µm; iv, Intravenous injection; HFHFHC, high fat, high cholesterol, high fructose. Statistical analysis was performed with one‐way ANOVA. *p < 0.05, **p < 0.01, ***p < 0.001 versus HFHFHC+AAV‐NC.

Patients with MASLD frequently present with hyperlipidemia, and the metabolic dysfunction associated with MASLD can further exacerbate dyslipidemia.^[^ ^18^ ^]^ Targeting pathways to reduce blood lipids, liver fat, and inflammation is a key approach to prevent and treat MASLD.^[^ ^19^ ^]^ To establish a clinically relevant MASLD model, we fed male APOE^(‐/‐)^ mice an HFHFHC diet for 12 weeks to induce liver lesions and hyperlipidemia.^[^ ^20^ ^]^ Simultaneously, hepatic asprosin was overexpressed in HFHFHC‐fed mice via tail vein injection of an AAV8 plasmid (**Figure** **4**a). Hepatic overexpression of asprosin was confirmed using Western blot and immunofluorescence (Figure S4a–c, Supporting Information). Significantly increased plasma levels of asprosin in hepatic asprosin overexpressing mice (Figure S4d, Supporting Information). After 12 weeks of HFHFHC feeding, the liver volume of APOE^(‐/‐)^ mice significantly increased, taking on a dark yellow appearance. In the HFHFHC+AAV‐Asprosin group, the liver showed further enlargement, swelling, and rounded edges (Figure S4e, Supporting Information). Doppler ultrasonography revealed an abnormal increase in the oblique diameter of the right liver lobe in APOE^(‐/‐)^ mice with hepatic overexpression of asprosin (Figure 4b, Figure S4f, Supporting Information). Both liver weight and LW/BW were significantly higher in the AAV‐Asprosin group compared to the model group (Figure 4c, Figure S4g, Supporting Information). Asprosin overexpression significantly elevated liver TC and TG levels compared to mice treated only with the HFHFHC diet (Figure 4d). Furthermore, serum levels of TC, TG, and LDL were significantly higher in asprosin‐overexpressing mice, while HDL levels further decreased (Figure 4e,f). Serum transaminase levels (ALT and AST) were also increased in hepatic asprosin overexpression mice, indicating more severe liver injury and lipid metabolism dysregulation (Figure 4g, Figure S4h, Supporting Information). Impaired glucose and insulin tolerance are contributors to insulin resistance in MASLD patients.^[^ ^21^ ^]^ OGTT and ITT tests showed that asprosin exacerbated HFHFHC‐induced insulin resistance (Figure 4h,i, Figure S4i,j, Supporting Information). Consistent with this, the serum glucose level was higher in the AAV‐Asprosin group than in the HFHFHC diet‐induced model group (Figure S4k, Supporting Information). AKT phosphorylation levels in response to insulin stimulation are positively correlated with insulin sensitivity in the liver. Hepatic overexpression of asprosin significantly reduced p‐AKT protein levels (Figure 4j). Histochemical and immunofluorescence staining showed that asprosin exacerbated HFHFHC diet‐induced hepatic steatosis, inflammatory infiltration, and hepatic fibrosis (Figure 4k–n, Supporting Information). Interestingly, asprosin significantly increased epididymal white fat and subcutaneous white fat area (Figure 4o), but had no significant effect on brown fat (Figure S4l, Supporting Information), indicating that asprosin promotes white fat hypertrophy and underscores its role as a critical factor in metabolic processes.

### 2.5 Transcriptome Analysis Reveals Asprosin Enrichment Pathway

**Figure 5:**
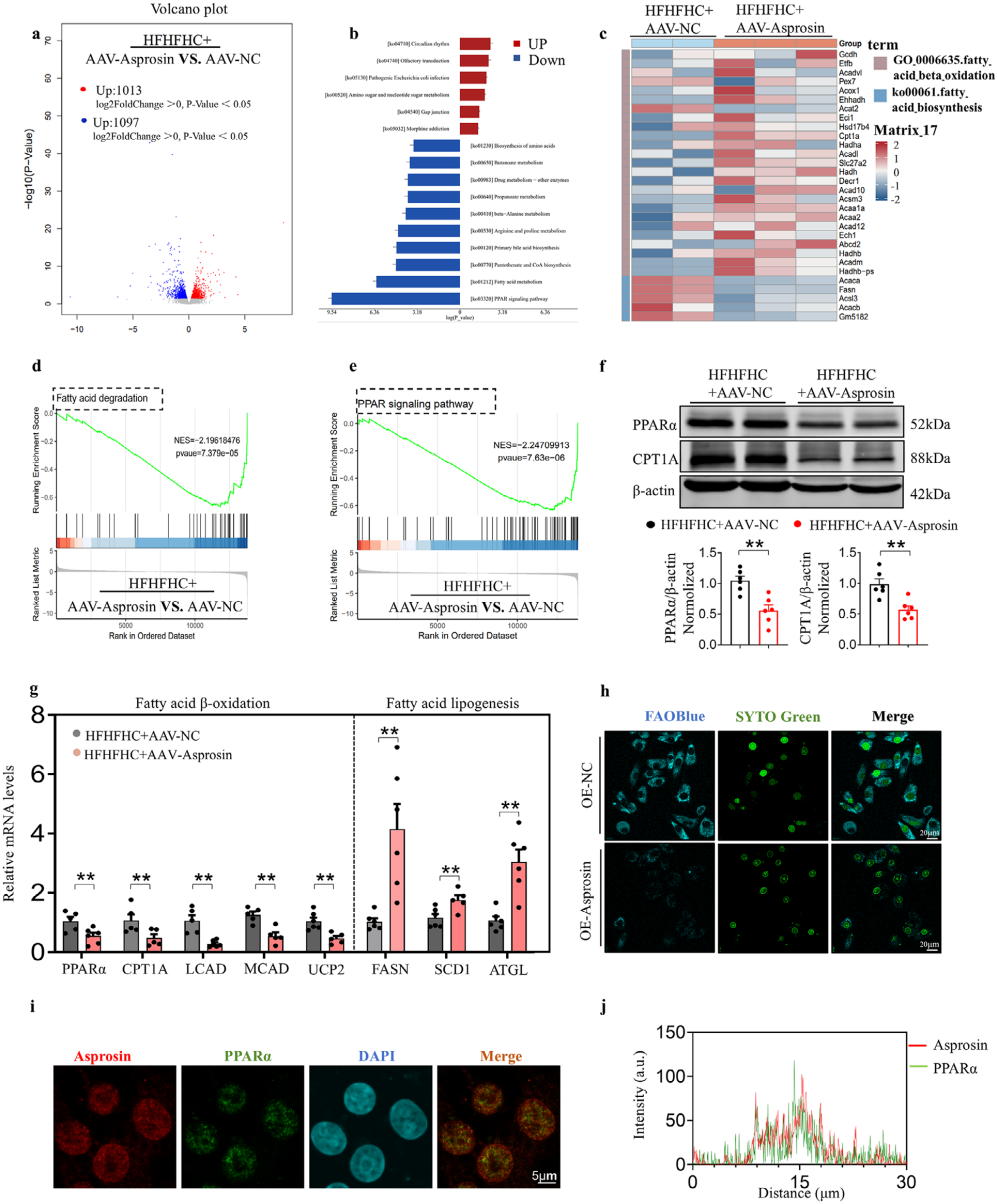
Transcriptome analysis reveals asprosin enrichment pathway. a–c) RNA sequencing conducted on liver tissues from mice fed an HFHFHC diet and treated with either AAV‐NC (control) or AAV‐Asprosin (asprosin overexpression). Top 15 pathways identified through KEGG enrichment analysis showing significant alterations. Heatmap depicting the expression levels of genes involved in fatty acid metabolism, demonstrating differential expression between groups. d,e) Gene Set Enrichment Analysis (GSEA) analysis of PPAR signaling pathway, fatty acid degradation pathway, indicating shifts in gene expression profiles related to lipid metabolism in response to asprosin modulation. f) Western blot detection of PPARα and CPT1A protein levels in liver. *n* = 6 in each group. g) Hepatic mRNA levels of genes related to lipid metabolism. *n* = 5–8 in each group. h) HepG2 cells underwent FAOBlue staining to assess β‐oxidation capacity. *n* = 5 in each group. i) Triple immunofluorescence (IF) staining for Asprosin (red), PPARα (green), and nuclei (DAPI, blue) in HepG2 cells. *n* = 6 in each group. j) Co‐localization analysis of PPARα with asprosin. Scale bar: 5 µm. Statistical analysis was performed with one‐way ANOVA. **p < 0.01 versus HFHFHC+AAV‐NC. HFHFHC, high fat, high cholesterol, high fructose.

To investigate the effects of asprosin on MASLD progression, we performed mRNA sequencing on liver samples from APOE^(‐/‐)^ mice fed an HFHFHC diet, with and without hepatic asprosin overexpression. The volcano plot revealed the downregulation of genes involved in fatty acid metabolism, such as CPT1A and ACOX1, in response to asprosin. Gene Set Enrichment Analysis (GSEA) identified significant downregulation of the PPAR signaling pathway and fatty acid degradation (**Figure** **5**a–e). PPARα plays a critical role in the liver by regulating mitochondrial fatty acid oxidation (FAO), inducing the transcription of fatty acid oxidases like carnitine palmitoyltransferase‐1 (CPT‐1).^[^ ^22^, ^23^ ^]^ Western blot results confirmed that hepatic asprosin overexpression significantly reduced PPARα and CPT1A protein levels (Figure 5f). In line with these findings, asprosin significantly increased the expression of genes involved in fatty acid lipogenesis and suppressed the expression of genes involved in FAO (Figure 5g). Additionally, asprosin reduced hepatocyte FAO capacity as demonstrated by live cell staining in HepG2 cells with FAOBlue (Figure 5h). Interestingly, immunofluorescence staining revealed that asprosin and PPARα predominantly colocalized in the nucleus in HepG2 cells (Figure 5i,j).

### 2.6 FABP5 Assists Asprosin in the Nuclear Translocation, and Asprosin Targets and Modulates the PPARα‐Binding PPRE Consensus Motif in CPT1A

**Figure 6:**
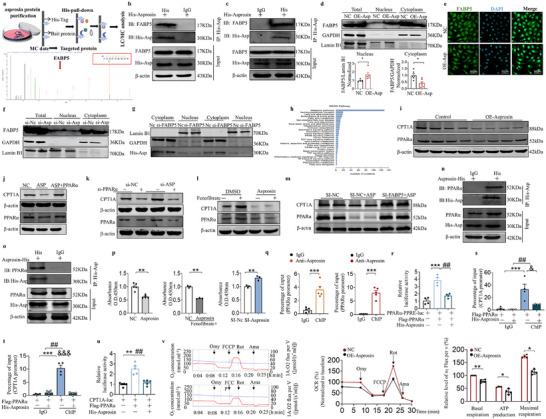
FABP5 assists asprosin in the nuclear translocation, and asprosin targets and modulates the PPARα‐binding PPRE consensus motif in CPT1A. a) Purification and interaction analysis of asprosin. His‐tagged asprosin protein was purified and subjected to a pull‐down assay in HepG2 cells to capture interacting proteins. Mass spectrometry identified specific peptides indicating high binding affinity between asprosin and FABP5. b,c) Interaction validation in primary hepatocytes and HepG2 cells. Cells were transfected with His‐asprosin, and immunoprecipitation (IP) using an anti‐His antibody confirmed the interaction between FABP5 and asprosin. *n* = 5 in each group. d) Asposin promoted the nuclear translocation of FABP5 in HepG2 cells. HepG2 cells were transfected with His‐asprosin before Western blot analyses. Statistical data of FABP5 protein expression. *n* = 5 in each group. e) Immunofluorescence visualization of nuclear translocation. HepG2 cells transfected with His‐asprosin were stained for FABP5 (green) and nuclei with DAPI (blue). *n* = 6 in each group. f) Asposin knock‐down reduces FABP5 nuclear translocation. Statistical data of FABP5 protein expression. *n* = 4 in each group. g) FABP5 knock‐down reduces asprosin nuclear translocation. *n* = 4, 5 in each group. h) Purification of asprosin protein and capture of interacting proteins using a His pull‐down assay in HepG2 cells, followed by KEGG pathway analysis of the identified proteins. i) Asprosin inhibited the expression of PPARα and its target gene CPT1A. Primary hepatocytes were treated with asprosin overexpression plasmid for 48 h and then analysed by Western blot. *n* = 8, 10 in each group. j) Asprosin reduced the expression and activity of PPARα, but cotransfected PPARα expression vector restored them. *n* = 6 in each group. k) Impact of PPARα‐targeting siRNA (siPPARα) on PPARα expression, and its effect on gene regulation by asprosin. Western blot analysis in primary hepatocytes transfected with siRNA or PPARα vectors. Western blot analysis of PPARα and CPT1A. *n* = 5, 6 in each group. l) Fenofibrate, a PPARα agonist, could not restore the effects of asprosin in primary hepatocytes treated with asprosin. After treatment with asprosin overexpression plasmid for 24 h, primary hepatocytes were treated with fenofibrate (100 µM) for 24 h. The protein level of PPARα was analyzed by Western blot. *n* = 6 in each group. m) Western blot analysis of the impact of FABP5 knockdown on asprosin's inhibition of PPARα and CPT1A protein expression in HepG2 cells. *n* = 4‐8 in each group. n,o) Primary hepatocytes and HepG2 cells were transfected with His‐asprosin. Immunoprecipitation (IP) was performed with an anti‐His antibody. IP assays showed that PPARα interacted with His‐asprosin in primary hepatocytes. *n* = 6 in each group. p) DNA‐binding activity of PPARα assessed using a transcription factor assay kit in HepG2 cells. (#ab133107, Abcam, USA). *n* = 3‐5 in each group. q). Chromatin immunoprecipitation (ChIP) analysis of asprosin binding to the PPARα promoters in primary hepatocytes and HepG2 cells. qPCR was performed with primers specific for the PPARα binding motifs. *n* = 5 in each group. r) HEK 293T cells were transfected with indicated plasmids. Cells were harvested after 24 h of transfection and luciferase activity was measured. *n* = 3 in each group. s,t) Chromatin immunoprecipitation (ChIP) analysis of PPARα binding to the CPT1A promoters in primary hepatocytes and HepG2 cells. qPCR was performed with primers specific for the CPT1A binding motifs. *n* = 4 in each group. u) HEK 293T cells were transfected with the indicated plasmids. Cells were harvested 24 h after transfected and the luciferase activity was measured. *n* = 6 in each group. v) Asprosin decreases mitochondrial oxygen consumption in HepG2 cells. ASP, asprosin. Statistical analysis was performed with one‐way ANOVA. *n* = 4–6 in each group. Statistical plot of basal, ATP‐linked and maximal respiration after treatment with asprosin overexpression plasmid for 48 h. Asp, asprosin. Scale bar: 50 µm. Statistical analysis was performed with one‐way ANOVA. *p < 0.05, **p < 0.01, ***p < 0.001 versus NC or control; ##*p* < 0.01 versus IgG or Flag‐PPARα+CPT1A‐luc; &*p* < 0.01 versus OE‐PPARα‐ChIP.

To investigate the target of asprosin and the reasons for its abnormal presence in the nucleus. We performed a proteomic screen using a histidine‐tag pull‐down assay combined with mass spectrometry analysis. First, asprosin was expressed in prokaryotes using the PET28a vector, and it was subsequently purified. Western blot analysis confirming the expression of asprosin protein is depicted in Figure S5a (Supporting Information). After successful protein purification (Figure S5b, Supporting Information), a His‐tag pull‐down assay was performed on HepG2 cells using asprosin purified protein. Mass spectrometry analysis identified 750 proteins potentially interacting with asprosin, and we focused on FABP5 for two reasons. First, FABP5 strongly binds to asprosin (**Figure** **6**a). Second, among the 750 proteins, only FABP5 binds hydrophobic ligands with varying affinities and transports these ligands and associated lipids to specific cellular compartments, including the nucleus and mitochondria.^[^ ^24^, ^25^, ^26^, ^27^ ^]^ To confirm the interaction between asprosin and FABP5, a plasmid encoding His‐asprosin was introduced into primary hepatocytes and HepG2 cells. Asprosin and FABP5 were effectively co‐immunoprecipitated (Figure 6b,c, Supporting Information). Since FABP5 can transport fatty acids into the nucleus, we speculated that asprosin could similarly enter the nucleus after binding to FABP5. Overexpression asprosin was detected in the nucleus of HepG2 cells through cytoplasmic nuclear extraction experiments (Figure S5c, Supporting Information). Although total FABP5 protein levels remained unchanged with asprosin overexpression, nuclear FABP5 levels significantly increased, while cytoplasmic FABP5 levels decreased (Figure 6d). The same results were also obtained by immunofluorescence in HepG2 cells and primary mouse hepatocytes (Figure 6e, Figure S5d, Supporting Information). Additionally, knockdown of asprosin produced the opposite results (Figure 6f), and nuclear asprosin levels were significantly reduced by FABP5 knockdown (Figure 6g, Figure S5e, Supporting Information). These results suggest that asprosin enters the nucleus through FABP5 transport without altering total FABP5 protein levels. It has been reported that asprosin regulates glucose homeostasis through OLFR734 receptor in liver. However, OLFR734 knockdown in HepG2 cells did not alter lipid accumulation induced by asprosin overexpression, as shown by Oil Red O staining (Figure S5f,g, Supporting Information), indicating that OLFR734 is not the target through which asprosin regulates lipid metabolism. To further verify whether asprosin regulates lipid metabolism through OLFR734 receptors in the liver, we generated plasmids targeting hepatocytes by liver‐specific siRNA OR4M1 plus N‐acetyl‐D‐galactosamine (GalNAc) modification. Liver‐specific overexpression of the AAV‐asprosin plasmid was first injected intravenously into the tail vein, followed by subcutaneous injection of GalNAc siRNA OR4M1 at a dose of 10 mg kg^−1^ at the end of the first week of the HFCDAA diet, and supplemented with additional injections at weekly intervals until sampling (Figure S5 h, Supporting Information). Western blot validated successful knockdown of hepatic OR4M1 protein and overexpression of asprosin protein (Figure S5i,j, Supporting Information). Hepatocyte‐specific overexpression of asprosin aggravated HFCDAA diet‐induced hepatic lipid accumulation and steatosis in mice, as shown by liver Oil red O and HE staining, whereas simultaneous knockdown of hepatic OR4M1 failed to reverse the effects of asprosin and the livers still contained a large number of lipid droplets and fat vacuoles (Figure S5k,l, Supporting Information). The same results were obtained for plasma transaminase (ALT, AST) levels in mice (Figure S5m,n, Supporting Information). Further support that OR4M1 (homolog of OLFR734) is not a key target of asprosin in the regulation of lipid metabolism. mRNA sequencing and KEGG analysis identified asprosin enrichment in the PPAR signaling pathway using pull‐down‐LS/MS‐MS (Figures 5e and 6 h). Immunofluorescence staining further demonstrated that asprosin and PPARα predominantly colocalize in the nucleus (Figure 5i,j). We validated that asprosin inhibited the expression of PPARα and CPT1A in HepG2 cells and primary mouse hepatocytes (Figure 6i, Figure S6a,b, Supporting Information), while co‐transfection with a PPARα overexpression plasmid restore the expression of PPARα and CPT1A (Figure 6j, Figure S6c–f, Supporting Information). Knockdown of asprosin increased PPARα expression, while PPARα‐targeting siRNA (siPPARα) inhibited this increase in PPARα and CPT1A expression (Figure 6k, Figure S6g,i, Supporting Information). We also found that asprosin inhibited the effect of PPARα agonist fenofibrate (Figure 6l, Figure S6h, Supporting Information). Moreover, FABP5 knockdown reversed the inhibitory effect of asprosin on PPARα and CPT1A protein expression (Figure 6 m, Figure S6j, Supporting Information). Immunofluorescence staining assay showed that asprosin and PPARα mainly colocalized in the nucleus. Co‐immunoprecipitation assays in mouse primary hepatocytes and HepG2 cells confirmed the specific interaction between asprosin and PPARα (Figure 6n–o). Moreover, asprosin significantly decreased the PPARα’s DNA‐binding activity in HepG2 cells (Figure 6p). Since PPARα is a transcription factor, the study of whether asproin is a transcriptional regulator. Our chromatin immunoprecipitation (ChIP) data showed that asprosin antibody significantly enriched PPARα in both primary mouse hepatocytes and HepG2 cells (Figure 6q). PPARα can bind to the PPRE consensus motif of target genes to modulate their expression. Luciferase reporter assay demonstrated that asprosin inhibited the transcriptional activity of PPARα to PPRE (Figure 6r). The CPT1A promoter region contains a PPARα‐binding PPRE consensus motif (Figure S6k, Supporting Information). ChIP enrichment assay found asprosin decreased the enrichment of PPARα in CPT1A promoter region in both primary mouse hepatocytes and HepG2 cells (Figure 6s,t). To further validate these results, We performed dual luciferase reporter assay using luciferase genes containing the CPT1A promoter region. Overexpression of asprosin significantly reduced the luciferase activity in Flag‐PPARα transduced HEK 293T cells (Figure 6u). Taken together, this suggests that asprosin targets and inhibits the binding of PPARα to the CPT1A promoter in primary mouse hepatocytes and HepG2 cells. CPT1A deficiency leads to mitochondrial respiratory dysfunction. As expected, overexpression of asprosin in HepG2 cells decreased the oxygen consumption rate, including basal respiration, ATP‐linked respiration, and maximal respiration (Figure 6v). The above data proves that FABP5 assists asprosin in the nuclear translocation in hepatocytes. In hepatocyte nucleus, asprosin targets and modulates the PPARα‐binding PPRE consensus motif in CPT1A, and inhibits hepatic mitochondrial fatty acid oxidation.

### 2.7 GalNAc‐siRNAs Targeting Hepatic FABP5 Alleviates Hepatic Steatosis Induced by HFHFHC Diet

**Figure 7:**
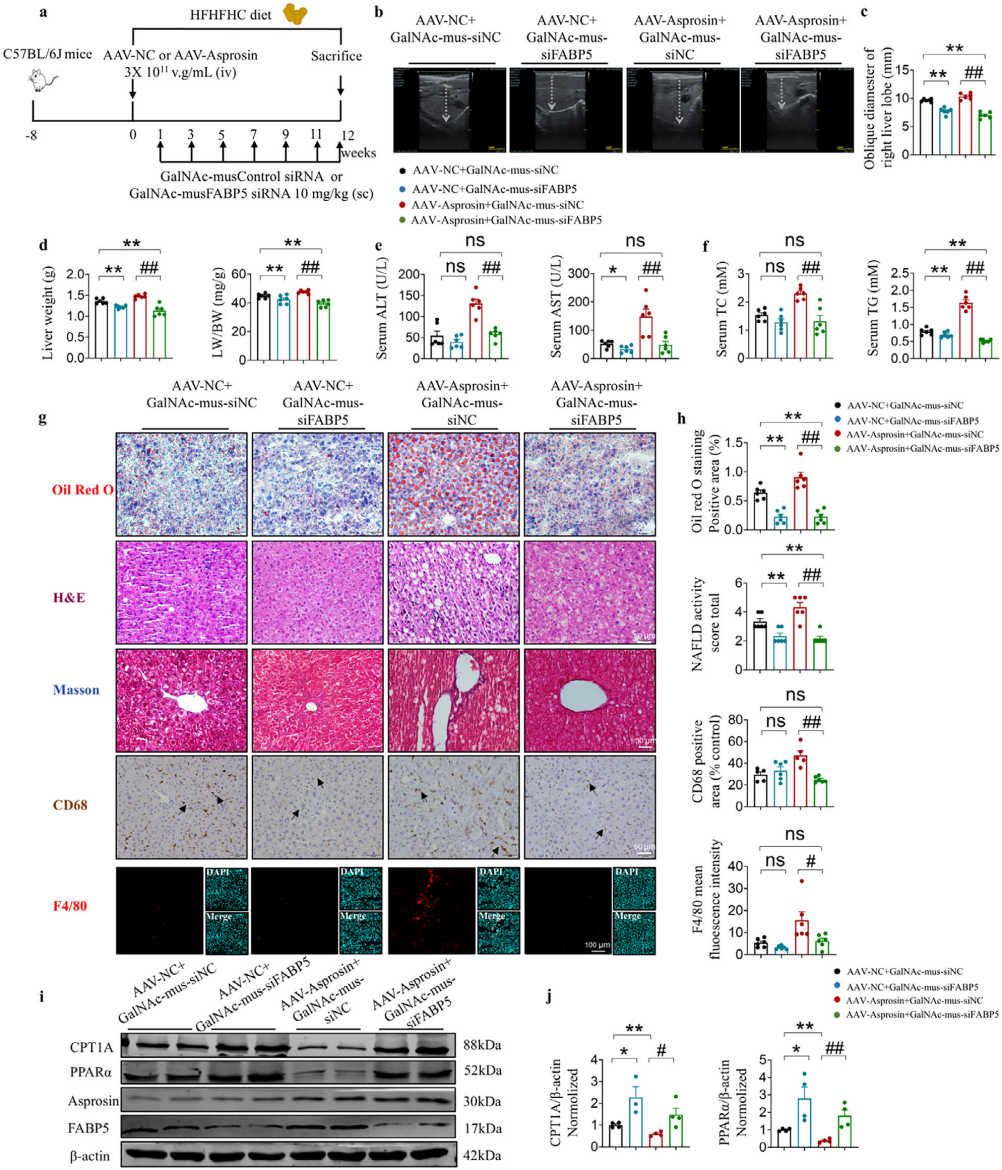
GalNAc‐siRNAs targeting hepatic FABP5 alleviate hepatic steatosis induced by HFHFHC diet. a) Schematic illustration of the experiment design, with an overview of GalNAc‐siFABP5 injection time points. b,c) The oblique diameter of the right lobe of the liver was detected by Doppler ultrasound. *n* = 6 in each group. d) The liver weight, and LW/BW of C57BL/6J mice from different groups. *n* = 6 in each group. e,f). Serum ALT, AST, TC, and TG levels in C57BL/6J mice. *n* = 6 in each group. g) Representative histological images of liver biopsies stained with Oil Red O, H&E, Masson's trichrome, F4/80 immunostaining, and CD68 immunohistochemical. *n* = 6 in each group. h) The statistics of Oil Red O‐positive areas, NAFLD activity score (NAS), quantitative of F4/80 and CD68 positive cells in liver. *n* = 6 in each group. i,j) Western blot detection of CPT1A, PPARAα, FABP5, asprosin protein levels in liver. *n* = 3,4 in each group. Scale bar for Oil Red O, H&E, Masson's trichrome, CD68 immunohistochemical staining: 50 µm, for F4/80 immunostaining: 100 µm. Statistical analysis was performed with one‐way ANOVA. *p < 0.05, **p < 0.01, ***p < 0.001 versus AAV‐shNC+GalNAc‐mus‐siNC. #*p* < 0.05, ##*p* < 0.01 versus AAV‐Asprosin+GalNAc‐mus‐siNC. ns, no significance.

To validate the results of the FABP5‐Asprosin interaction in regulating PPARα activity and mitochondrial fatty acid oxidation in hepatocytes, we generated a plasmid targeting hepatocyte to knock down of FABP5 by liver‐specific siRNA FABP5 plus N‐acetyl‐D‐galactosamine (GalNAc) modification, injected at 10 mg kg^−1^ via subcutaneous injection at the end of the first week HFHFHF diet administration, and supplemented with injections at one‐week intervals until sampling (**Figure** **7**a). GalNAc‐siFABP5 treatment significantly reduced right lobe diameter, liver weight, and liver weight index induced by HFHFHC diet, The increased right lobe oblique diameter and liver weight index caused by hepatocyte‐specific overexpression of asprosin were reversed by GalNAc‐siFABP5 (Figure 7b–d). GalNAc‐siFABP5 treatment reduced serum AST, ALT, TC, TG levels (Figure 7e,f), attenuated hepatic steatosis and fibrosis progression, and lowered inflammation and NAS scores. Similarly, the exacerbation of hepatic steatosis, inflammation, and fibrosis induced by hepatocyte‐specific overexpression of asprosin in HFHFHC mice was also rescued by hepatocyte‐specific knockdown of FABP5 (Figure 7g,h). Consistent with the results of the in vitro experiments (Figure 6 m), GalNAc‐siFABP5 treatment reversed asprosin‐induced inhibition of PPARα and its target protein CPT1A (Figure 7i,j and Figure S7a,b, Supporting Information). Taken together, these results suggest that FABP5‐asprosin interaction promotes hepatic steatosis, inflammation, and fibrosis progression by inhibiting PPARα activity in mice.

### 2.8 AAV‐shAsprosin Enhances Effects of Fenofibrate in HFCDAA‐fed Mice

**Figure 8:**
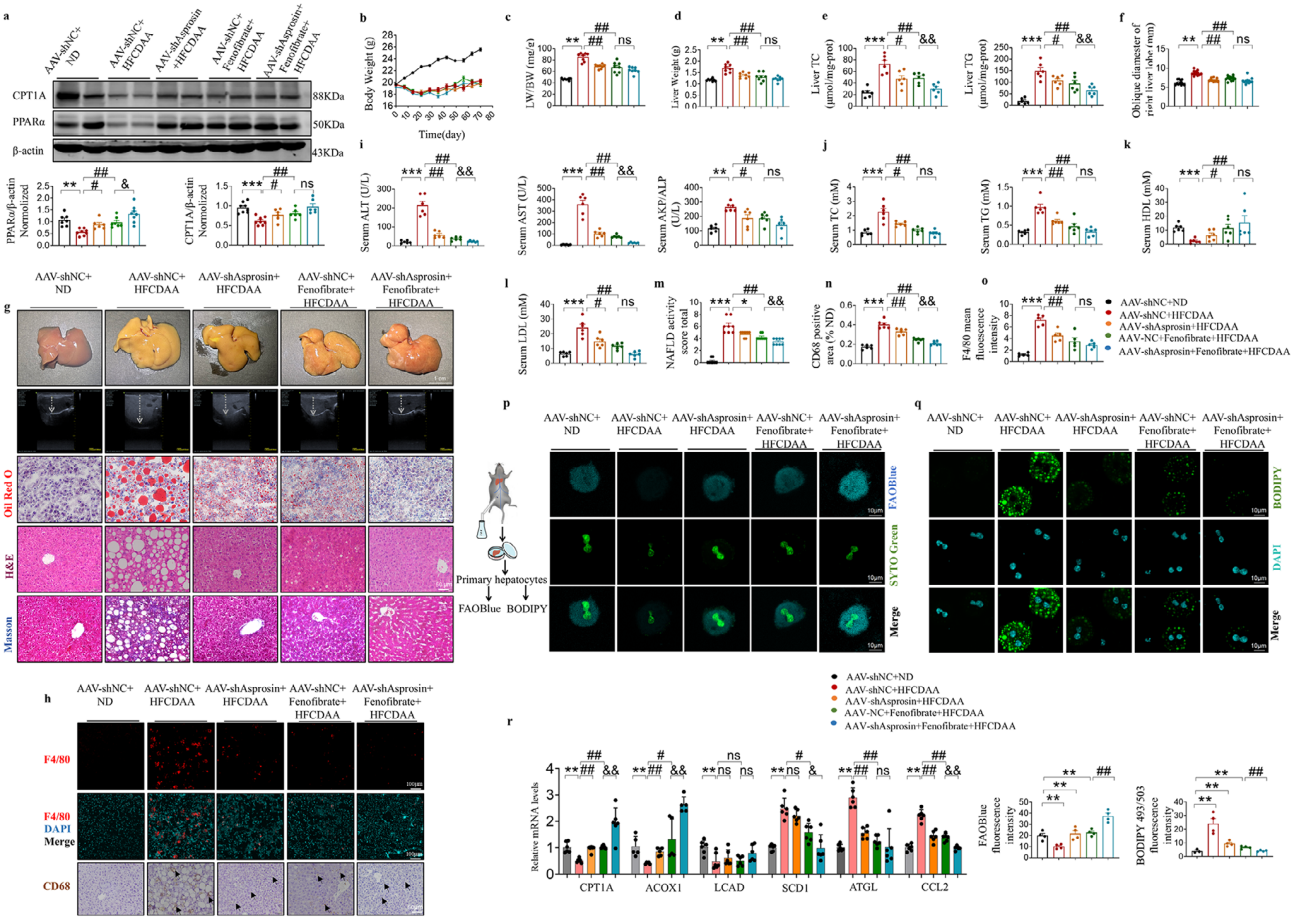
AAV‐shAsprosin enhances the effects of fenofibrate in HFCDAA‐fed mice. a) Western blot detection of PPARα and CPT1A protein levels in liver. *n* = 6 in each group. b–o) Statistical images of the oblique diameter of the right lobe of the liver detected by Doppler ultrasound; The body weight, liver weight, and LW/BW of C57BL/6J mice; The TC and TG content of livers; Plasma biochemical tests. Statistical analysis was performed with one‐way ANOVA. *n* = 6‐8 in each group. ALT, aspartate aminotransferase; AST, alanine aminotransferase; AKP, alkaline phosphatase; TC, total cholesterol; TG, triglyceride; HDL‐C, high‐density lipoprotein cholesterol; LDL‐C, low‐density lipoprotein cholesterol; LW/BW, liver weight/body weight; ND, normal diet; HFCDAA, high fat, methionine choline deficiency diet. Statistics of Oil Red O positive areas, NAFLD activity score (NAS), quantitative of F4/80 and CD68 positive area in the liver are shown. *n* = 6, 7 in each group. g) C57BL/6 J mice were fed on HFCDAA and treated with the indicated AAVs. After C57BL/6 J mice were fed on HFCDAA for 4 weeks, mice were injected with vehicle or fenofibrate (100 mg kg^−1^) for 4 weeks. Representative images, the oblique diameter of the right lobe of the liver detected by Doppler ultrasound, Oil Red O, H&E, and Masson's trichrome. *n* = 6–8 in each group. h) Representative images of liver biopsies stained for F4/80 immunofluorescence and CD68 immunohistochemistry. p,q) Schematic diagram of primary hepatocytes extracted for subsequent experiments. Primary hepatocytes, isolated from the livers of treated mice, underwent FAOBlue staining to assess β‐oxidation capacity, and BODIPY staining to evaluate lipid deposition. The statistics of quantification were evaluated by FAOBlue staining and BODIPY staining. *n* = 4 in each group. HFCDAA, high fat, methionine choline deficiency diet. r) q‐PCR was performed to use the hepatic mRNA levels of genes related to fatty acid metabolism. *n* = 5,6 in each group. Scale bar for Oil Red O, H&E, Masson's trichrome staining, and CD68 immunohistochemistry staining: 50 µm, for FAOBlue and BODIPY staining: 10 µm, Scale bar for F4/80 immunofluorescence staining: 100 µm. Statistical analysis was performed with one‐way ANOVA. **p < 0.01,***p < 0.001 versus AAV‐NC+ND; # *p* < 0.05; ##*p* < 0.01 versus AAV‐NC+HFCDAA; &&*p* < 0.01 versus AAV‐NC+Fenofibrate+HFCDAA; ns, not significant. ND, normal diet; HFCDAA, high fat, methionine choline deficiency diet.

Recently, drug development strategies are shifting toward combination therapy. Since asprosin inhibited the PPARα activating effect of PPARα agonist fenofibrate (Figure 6k, Figure S6h, Supporting Information). We investigated whether the combination of AAV‐shAsprosin and the PPARα agonist fenofibrate could further improve MASH in HFCDAA‐fed mice. As expected, co‐administration increased PPARα levels more than fenofibrate alone (**Figure** **8**a). AAV‐shAsprosin or fenofibrate alone could reduce the liver weight, LW/BW, and hepatic TG and TG levels, which were further improved by concomitant treatment (Figure 8b–e). Furthermore, the combination treatment significantly improved hepatic steatosis, reduced the progression of hepatic fibrosis and the generation of inflammation (Figure 8f,h,m, decreased the elevation of abnormal serum transaminase levels (Figure 8i), and liver appearance and dyslipidemia were greatly improved, with the liver color (Figure 8g ), and blood lipid concentrations returning to near normal (Figure 8j,k). CD68 immunohistochemistry and F4/80 immunofluorescence revealed that combination therapy significantly reduced liver inflammation (Figure 8h,n,o). Meanwhile, FAOBlue staining for FAO in living cells and BODIPY staining for lipid accumulation performed on primary hepatocytes from all mouse groups showed that AAV‐shAsprosin combined with fenofibrate therapy significantly increased FAO in the mitochondria of primary hepatocytes and significantly reduced lipid deposition which was more effective than either treatment alone (Figure 8p,q). Additionally, the expression of genes involved in lipids synthesis, such as ATGL, SCD1, and liver fibrosis, such as CCL2 decreased with co‐administration. The expression of genes related to FAO, such as CPT1A, ACOX1, LCAD increased (Figure 8r). These findings suggest that AAV‐shAsprosin can compensate for the limitations of fenofibrate in the treatment of MASH, and that inhibition of hepatic asprosin may be an effective option for the treatment of MASLD/MASH.

## 3 Discussion

Hepatic steatosis is the precursor of hepatic fibrosis and cirrhosis, conditions that result from the sustained presence of hepatic steatosis. However, the key molecules and underlying signaling pathways involved in this process remain incompletely understood. This study elucidates the pathophysiological role of asprosin and delineates its underlying mechanisms in greater detail in the context of MASLD. We observed that circulating asprosin levels were significantly higher in MASLD patients compared to healthy controls. Furthermore, our data demonstrated that asprosin levels markedly increased in the livers of several MASLD mouse models, contributing to disease progression. This was further supported by the observation that hepatocyte‐specific asprosin knockdown alleviated hepatic steatosis, inflammation, and fibrosis in an HFCDAA diet‐induced mouse model, while liver‐specific asprosin overexpression via adeno‐associated virus delivery exacerbated hepatic steatosis, liver injury, and dyslipidemia. Furthermore, hepatocyte‐specific knockdown of FABP5 significantly reversed asprosin‐exacerbated hepatic steatosis in HFHFHC mice. Surprisingly, white adipose tissue, the previously reported source of asprosin, specific knockdown of asprosin failed to alleviate hepatic steatosis and dyslipidemia in the HFCDAA diet‐induced model, suggesting that therapeutic interventions targeting asprosin for the treatment of MASLD/MASH should specifically target hepatocytes rather than adipose tissue.

Asprosin has been shown to have multiple effects on different tissue types. Asprosin weakens insulin signaling in skeletal muscle and induces insulin resistance via inflammatory mechanisms.^[^ ^28^, ^29^ ^]^ Moreover, it amplifies inflammation in both endothelial cells and macrophages.^[^ ^30^, ^31^ ^]^ However, one study has reported that asprosin mitigates the progression of atherosclerosis.^[^ ^32^ ^]^ Given these contradictory findings and the absence of correlational studies, it is imperative to conduct in‐depth research on the precise role of asprosin in metabolic processes. Clinical studies have strongly associated circulating asprosin levels with disease severity in conditions such as obesity, MASLD, and other obesity‐related cardiometabolic conditions.^[^ ^33^, ^34^ ^]^ By studying MASLD patients and mice, we confirm that plasma asprosin levels are positively correlated with markers of liver injury as well as TC and TG.

mRNA sequencing analysis of livers from mice with liver‐specific overexpression of asprosin reveals significant inhibition of the PPARα signaling pathway. Additionally, asprosin is found to co‐localize with PPARα in the nucleus of HepG2 cells. To investigate asprosin's molecular target and the mechanisms underlying its abnormal nuclear presence, we have performed a proteomic screen using histidine‐tag pull‐down and mass spectrometry analysis. FABP5, which binds strongly to asprosin, is identified as a key protein in the mass spectrometry results of the His pull‐down assay. As a small molecule intracellular soluble protein, FABP5 can transport fatty acids into the nucleus and, in specific conditions, modulate the activity of certain transcription factors.^[^ ^23^, ^24^ ^]^ In prostate cancer, FABP5 transports fatty acids to nuclear receptors, thereby promoting tumor metastasis in a process called FABP5 nuclear translocation.^[^ ^26^ ^]^ This process facilitates the entry of certain factors into the nucleus, where they interact with nuclear substances.^[^ ^35^, ^36^ ^]^ Since FABP5 can ectopically transport fatty acids into the nucleus, we hypothesize that asprosin enters the nucleus with the assistance of FABP5. As expected, our experiments have demonstrated that while asprosin does not affect the total protein level of FABP5, it does bind to FABP5 and translocates it to the nucleus. Asprosin inhibits the expression of FAO‐related genes by repressing PPARα transcriptional activity, ultimately impairing FAO and resulting in abnormal mitochondrial function in hepatocytes.

Given the strong causal link between cardiovascular risk and MASLD, therapeutic approaches targeting glucose and lipid metabolism have yielded promising outcomes, including insulin sensitizers, adipogenesis inhibitors, fatty acid oxidation activators, and PPAR α/γ/δ agonists. The lipid‐regulating agent fenofibrate is currently under evaluation for the treatment of MASLD. Animal studies have proved that fenofibrate improves ALT and AST levels and reduces hepatocyte ballooning degeneration, while its effect on histological endpoints of hepatic steatosis is still being investigated. Here, we demonstrate that increased hepatic asprosin levels in MASLD suppress PPARα expression, which may explain fenofibrate's limitations in treating MASLD. The secretory adipose factor asprosin is expected to act as a biological marker for early clinical diagnosis and prognostic evaluation of MASLD. Moreover, targeting hepatic asprosin gene inhibition and GalNAc‐siRNAs to inhibit hepatic FABP5 both offer potential therapeutic benefits in the management of MASLD. Asprosin inhibition can enhance the effects of PPARα agonists, and their combination could provide a more effective strategy for managing MASLD/MASH.

## 4 Experimental Section

### Cell Culture and Reagents

HepG2 cells and HEK 293T cell line were purchased from Shanghai Zhong Qiao Xin Zhou Biotechnology (Shanghai, China). Cells were cultured in Dulbecco's Modified Eagle's Medium supplemented with 10% fetal bovine serum (FBS, Biological Industries, Israel) and 1% penicillin and streptomycin.

Mouse primary hepatocytes (MPHs) were isolated from the livers of male C57BL/6J mice using standard procedures. Mice were anaesthetized and perfused via portal vein with freshly prepared followed by digest buffer (collagenase IV, Worthington, NJ). Subsequently, the perfused livers were excised. Viability was determined by trypan blue exclusion assay (Corning, Rochester, NY). MPHs were cultured in DMEM supplemented with 10% fetal bovine serum, 10 mM insulin (HY‐P0035, MCE, USA), and 10 mM dexamethasone (HY‐14648, MCE, USA). All antibodies used were validated, as evidenced by cited references.

Fibrillin‐1/asprosin protein, human (HEK293, His, HY‐P7612, USA) was used. Fenofibrate was obtained from MCE (HY‐17356, USA), The antibodies used for Western blot included: PPARα (66826‐1‐1 g, Proteintech, USA), CPT1A (15184‐1‐AP, Proteintech, USA), FABP5 (12348‐1‐AP, Proteintech, USA), Lamin B1 (#A1910, Abclonal, Wuhan, China), β‐Actin (#AC026, Abclonal, Wuhan, China), GAPDH (#AC002, Abclonal, Wuhan, China), P‐AKT (ser473) (#4060, CST, USA), T‐AKT (#9272, CST, USA), goat anti‐rabbit IgG (#926‐32211, LI‐COR Biosciences, USA), Asprosin (#FNab09797, FineTest, Wuhan, China), His (#66005‐1‐1, Proteintech, USA), Flag (#66008‐1‐1 g, Proteintech, USA). FBN1 (#YT1684, ImmunoWay, China), PPARα antibodies used included #GTX22779 (GeneTex, USA) for ChIP experiments and #ab61182 (Abcam, UK) for immunofluorescence studies.

### Animal Maintenance and Experiments

All animal care and experimental protocols for this study were in accordance with the Chinese Regulations on the Management of Laboratory Animals and were approved by the IRB of the College of Pharmacy, Harbin Medical University (project license number: IRB5064724). Animal studies were conducted in compliance with the ARRIVE guidelines.^[^ ^37^, ^38^ ^]^

Specific pathogen‐free (SPF) male C57BL/6J mice (20–23 g, 8 weeks old), SPF male Syrian hamster (100–110 g, 6 weeks old) and SPF male ApoE^(‐/‐)^ mice (23–27 g, 8 weeks old) mice were obtained from Changsheng biotechnology (Liaoning, China). All animals were kept in a pathogen‐free environment with controlled temperature (22 ± 2 °C) and a 12 h light/dark cycle. They had ad libitum access to food and water throughout the study period. All animals in this study were fasted for 6 h and then executed for subsequent experiments.

C57BL/6J mice were fed the HFHFHC diet for 12 consecutive weeks to establish a mouse model hepatic steatosis. Mice fed a normal diet (ND) served as controls. ApoE^(‐/‐)^ mice, Hamsters, and C57BL/6J mice were fed a HFD for 21 weeks, 4 weeks, and 12 weeks, respectively, to induce hepatic steatosis.

C57BL/6J mice were divided into the following grouping for further study: those on a normal diet supplemented with AAV8‐shNC serving as the control group, and those on an HFCDAA diet receiving tail vein injections of AAV8‐shNC or AAV8‐shAsprosin (Genechem, Shanghai, China) for specific knockdown of hepatic asprosin. All viral injections were administered one week after the acclimatization of the mice.

To generate adipose tissue‐specific asprosin knockout mice, C57BL/6J mice were intraperitoneally injected with AAV8 encoding the FBN1 gene under the hAdipoq mini promoter. Adeno‐associated viruses were produced by Cyagen Biosciences (Guangzhou, China).

Another set of ApoE^(‐/‐)^ mice was divided into the following grouping: each fed the HFHFHC diet and receiving tail vein injections of either AAV8‐NC or AAV8‐Asprosin for specific overexpression of hepatic asprosin.

To generate a plasmid targeting hepatocyte knockdown of FABP5 by liver‐specific siRNA FABP5 plus N‐acetyl‐D‐galactosamine (GalNAc) modification. 10 mg kg^−1^ was injected via subcutaneous injection during the first week of administration of the HFHFHF diet, and supplemented with injections at one‐week intervals until sampling.

C57BL/6J mice were also fed an HFCDAA diet for 8 consecutive weeks to establish another model of hepatic steatosis: one on an HFCDAA diet alone, another supplemented with AAV8‐shNC injections, a third receiving both AAV8‐shNC injections and fenofibrate gavage (100 mg kg^−1^ day^−1^) for 4 weeks, a fourth with AAV8‐shAsprosin injections for specific hepatic asprosin knockdown, and wild‐type (WT) mice on a normal diet with AAV8‐shNC injections serving as controls.

### MASLD Human Patients Study

All experiments were approved by the IRB of the College of Pharmacy, Harbin Medical University (project license number: IRB5064724). MASLD patients and healthy controls were provided by the Second Affiliated Hospital of Harbin Medical University. The patients all signed an informed consent form. The inclusion criteria for patients with MASLD were 1) exclusion of excessive alcohol consumption, with weekly ethanol intake ≥210 g in men and ≥140 g in women, 2) exclusion of other causes that can lead to fatty liver such as viral hepatitis. 3) Liver ultrasonography suggesting hepatic steatosis and at least 1 metabolic cardiovascular disease risk factor, such as fasting blood glucose ≥6.1 mmol L^−1^ and fasting triglycerides ≥1.7 mmol L^−1^. All participants complied with the rules guiding human experimentation in the Declaration of Helsinki and gave informed consent in writing.

### Asprosin Enzyme‐Linked Immunosorbent Assay

Commercially available mouse asprosin ELISA kits (#EM1707, FineTest, Wuhan, China) were utilized to quantify asprosin levels in serum and cell lysates, following the manufacturer's instructions. Commercially available human asprosin ELISA kits (#EH4176, FineTest, Wuhan, China) were utilized to quantify asprosin levels in human plasma.

### Western Blotting

Protein samples were obtained using RIPA buffer. After quantification of proteins with the BCA Protein Detection Kit. Primary antibodies were incubated overnight at 4 °C, followed by 1 h of fluorescence‐labeled secondary antibodies. Images were acquired using the Odyssey CLx Infrared Imaging System.

### RNA Isolation and Real‐Time Quantitative RT‐PCR

Total RNA was isolated from cultured HepG2 cells, MPHs, and mouse liver tissues using TRIzol and the Concentration of the extracted RNA was measured with a NanoDrop 8000 (Thermo, USA). Quantitative real‐time PCR assays were performed on an Applied Biosystems 7500 Fast Real‐Time PCR System. (Applied Biosystems, USA). Primer sequences are shown in Table S3 (Supporting Information).

### Oil red O staining and BODIPY staining

Cultured HepG2 cells and mouse primary hepatocytes were grown to 70% confluence. An in vitro cellular lipotoxicity model was established by treating the cells with 250 µM FFA for 24 h. Depending on the experimental group, cells were transfected with the asprosin overexpression plasmid or siRNA targeting asprosin for 48 h, or treated with asprosin recombinant protein for 24 h. Cells were stained with Oil Red O (#G1261, solarbio, China) for 15 min or BODIPY (#GC42959, GlpBio, USA) for 30 min. Images were captured using Leica microsystems (Leica, Solms, Germany) or observed under a fluorescence microscope (BX53, OLYMPUS, Japan).

### Construction and Transfection of siRNA

siRNAs targeting asprosin, FABP5, OFRL734, and PPARα were designed and synthesized by RiBobio (Guangzhou, China). Overexpression plasmids for PPARα and asprosin were produced by HonorGene (Changsha, China). HepG2 cells and MPHs were inoculated in six‐well plates. Six‐well plates were spiked with 250 µL of siRNA‐Lipofectamineo 2000 mixture per well. Incubate the transfection for 6 h. The cells were then incubated for a further 48 h in serum containing medium. Subsequently, the cells were prepared for subsequent experiments. The sequences of the above genes are as follows:

Human asprosin siRNA: 5′‐GTGATAATCTGAAGATGAA‐3′

Mouse asprosin siRNA: 5′‐GTACATCGGCACTCACTGT‐3′

Human PPARα siRNA: 5′‐ GGAGCATTGAACATCGAAT‐3′

Mouse PPARα siRNA: 5′‐CTCCTCGGTGACTTATCCT‐3′

Human FABP5 siRNA: 5′‐CGGATCTATGAAAAAGTAG‐3′

Mouse FABP5 siRNA: 5′GCCAAGCCAGACUGUAUCATT‐3′

Human OLFR734 siRNA: 5′‐GGAAUGGUAUCUGGUAAACAUTT‐3′

Mouse OLFR734 siRNA: 5′‐GUUGGCUCAGUGGAAUGGUAUTT‐3′

### Adeno‐Associated Virus Injection

An rAAV system (type 8) expressing scramble shRNA and asprosin shRNA was used to manipulate the expression of asprosin in vivo. Adeno‐associated virus by tail vein injection. Adeno‐associated viruses were supplied by Shanghai GeneChem Medical Technology Co., Ltd. Asprosin shRNA was constructed in a miRNA (e.g., miR155) backbone by assembling three target sequences in a tandem fashion. The three sequences are as follows:

1. AAATGAAGGAGCCAGGGCTGT
2. ATAAATGTCAATCACGAAGCC
3. AAATCTTGGCACCTTCTTCCA

Adeno‐associated virus 8 (AAV8) encoding mouse FBN1 gene under the hAdipoq mini promoter. The viral dose administered to each mouse was 2.0 × 10¹¹ GC mL^−1^, a concentration optimized for efficient gene knockdown in adipose tissue.

Knockdown of OR4M1 in liver by siRNA OR4M1 targeting, Introducing GalNAc modifier gene in siRNA OR4M1 molecule to improve siRNA targeting to liver. 10 mg kg^−1^ was injected via subcutaneous injection, and supplemented with injections at one‐week intervals until sampling. GalNAc‐musOR4M1 siRNA were generated by Changsha Abiowell Biotechnology Co., Ltd. Mouse OR4M1 siRNA: 5′‐GCCCAAUGAACUAGACAAUTT‐3′.

Knockdown of FABP5 in the liver by siRNA FABP5 targeting, Introducing GalNAc modifier gene in siRNA FABP5 molecule to improve siRNA targeting to the liver. About 10 mg kg^−1^ was injected via subcutaneous injection, and supplemented with injections at one‐week intervals until sampling. GalNAc‐musFABP5 siRNA were generated by Changsha Abiowell Biotechnology Co., Ltd. Mouse FABP5 siRNA: 5′GCCAAGCCAGACUGUAUCATT‐3′.

### Liver Ultrasonography

Inhalation of isoflurane induced anaesthesia in the animals. The oblique diameter was measured using a Doppler ultrasound diagnostic system (VINNO Technology, Suzhou, China).

### OGTT and ITT

After 10 weeks on the HFHFHC diet, oral glucose tolerance tests (OGTT) were performed on mice after an 8‐h fast. Blood glucose levels were recorded at baseline, 15, 30, 60, and 120 min after oral administration of 0.5 g mL^−1^ of D‐glucose solution (Sigma‐Aldrich, USA) to mice. The insulin tolerance test (ITT) was conducted 1 week after the OGTT.

### Hepatic Lipid Analysis

Liver tissue is lysed using the lysis buffer provided in the kit (#E1025‐105T, #E1026‐105T, Applygen, Beijing, China). The supernatant was analyzed for TG and TC concentrations after centrifugation at 2000 g for 5 min.

### Serum Assays

The serum was isolated by centrifugation (4000 g, 15 min) from blood samples that had been allowed to rest for 4 h at room temperature. Serum aminotransferase levels and lipid levels were then measured.

### Hydroxyproline Assay

Hepatic collagen content was assessed by quantifying hydroxyproline levels in the liver using the Hydroxyproline Assay Kit (#BC0255, Solarbio, Beijing, China). Liver samples were hydrolyzed in concentrated hydrochloric acid at 120 °C for 3 h and other reagents were added as required.

### Histopathologic Analysis

Liver paraffin sections were stained with hematoxylin and eosin (H&E) or Masson's staining, and the white adipose tissue sections and brown adipose tissue were also stained with H&E. Additionally, frozen sections of liver were stained with Oil Red O to assess lipid deposition. Immunohistochemistry was performed using CD68 antibody to assess macrophage content.

### Immunofluorescence

HepG2 cells and mouse primary hepatocytes were cultured on glass dishes. Depending on the experimental group, cells were transfected with asprosin overexpression plasmid or asprosin shRNA plasmid. FABP5 antibody (dil:1:200, #12348‐1‐AP, Proteintech, USA) incubated with cells at 4 °C overnight. Then incubation with Alexa Fluor 488‐conjugated secondary antibody (dil:1:300, #p0188, Beyotime, China) for 1 h to detect the nuclear translocation of FABP5.

For double staining, sections were incubated overnight at 4 °C with asprosin antibodies (#FNab09797, FineTest, Wuhan, China), and PPARα antibodies (#66826‐1‐Ig, Proteintech, USA), followed by a 30‐min incubation with Alexa Fluor 594 (rabbits) and Alexa Fluor 488 (mice) (Thermo Fisher, USA) ‐conjugated secondary antibodies. Frozen section of the liver after fixation and blocking, the slides were incubated with the following primary antibodies: F4/80 (1:200) (#DF2789, Affinity Biosciences, Nanjing, China), asprosin (1:200) (#FNab09797, FineTest, Wuhan, China) and His antibody (#66005‐1‐1 g, Proteintech, USA). The subsequent secondary antibodies were TRITC (red) and FITC (green) (Proteintech, USA) for 1 h at room temperature, followed by counterstaining with DAPI (#G1012, Servicebio, China) was used for nuclear staining. Analyzed using a fluorescence microscope (Olympus, Tokyo, Japan).

### PPARα DNA Binding Activity Assay

PPARα DNA‐binding activity was quantitatively assessed using the PPARα Transcription Factor Assay Kit (#ab133107, Abcam, UK). The test was carried out by the addition of specific primary antibodies, these were followed by horseradish peroxidase‐conjugated secondary antibodies, in accordance with the kit's instructions. The reaction was developed using the appropriate solutions, and the absorbance was measured to determine the binding activity of PPARα.

### Luciferase Assay

Luciferase reporter assays were conducted to evaluate the transcriptional activity of the PPAR response element and the CPT1A promoter. Reporter plasmids pGL3‐basic‐CPT1Ap and pGL3‐basic‐PPRE, containing the CPT1A promoter and PPAR response element respectively, were acquired from HonorGene (Changsha Abiwei Biotechnology Co., Ltd.). These plasmids utilize the pGL3‐basic vector from Promega with the respective promoter fragments inserted.

For the dual‐luciferase reporter assays, DNA transfection was performed using the specified reporter plasmids alongside the Renilla luciferase plasmid (pRL‐TK) as a control for normalization. Cells were co‐transfected with the respective luciferase reporters and either Flag‐tagged PPARα alone or combined with His‐tagged asprosin. Luciferase activities were quantified 24 h post‐transfection using the BioTek Synergy HTX multimode reader, where firefly luciferase activity was normalized against Renilla luciferase activity from the same sample to adjust for transfection efficiency. Specifically, the effects of asprosin on PPARα ‐regulated transcription of the target gene CPT1A were assessed by measuring the activity in cells transfected with the CPT1A‐Luc vector and the PPRE‐Luc vector under the influence of Flag‐PPARα, with and without His‐asprosin co‐expression.

### Chromatin Immunoprecipitation (ChIP) Assay

Chromatin immunoprecipitation (ChIP) assays were conducted as per the manufacturer's protocol (#26 157, Thermo Fisher Scientific, USA). To cross‐link DNA‐protein complexes, the cells were first fixed with 1% formaldehyde in a culture medium for 10 minutes at room temperature. Following fixation, cells were collected and lysed, and the chromatin was digested with micrococcal nuclease (MNase) to generate sheared chromatin fragments. Immunoprecipitation was carried out using an anti‐PPARα antibody or a normal rabbit IgG as a negative control, overnight at 4 °C with constant rotation.

Protein‐DNA complexes were captured using protein A/G agarose beads. These complexes were then eluted using a high‐salt buffer at 4 °C. The DNA was purified from the eluted complexes and subjected to quantitative real‐time PCR (qPCR) to assess the enrichment of PPARα binding at the CPT1A promoter region. The qPCR employed specific primers designed to amplify a 383 bp fragment encompassing the PPRE sequence within the CPT1A promoter.

F 5′‐CCCACGTGCAAGGAGAGC‐3′ R 5′‐CAGGCACGTGCGACATTTTG‐3′

The PPARα promoter fragment:

F 5′‐GCATGTGAAGGCTGTAAGGG‐3′ 5′‐TTGTGTGACATCCCCGACAGA‐3′

### Co‐Immunoprecipitation Assay

Co‐immunoprecipitation assays were tailored based on experimental subgroups. In primary hepatocytes and HepG2 cells, the His‐asprosin overexpression plasmid was transfected and maintained for 48 h. Following transfection, cells were lysed using IP lysis buffer. For antibody immobilization, 20 µg of His‐asprosin antibody was used per reaction. 500 µL of pre‐washed cell lysate was added to the antibody‐coated centrifuge column and incubated at 4 °C overnight. Followed by the addition of Elution Buffer to further elute bound complexes. To assess the interaction between His‐asprosin and Flag‐PPARα, the proteins were analyzed by Western blot.

### Mitochondrial Respiratory Measurement

Mitochondrial respiratory function of HepG2 cells was assessed following an established protocol using high‐resolution respirometry (Oxygraph‐2k, Oroboros Instruments, Innsbruck, Austria). The measurement commenced with the recording of routine respiration rates. The respirometry was then adjusted to a non‐phosphorylating leak state through the addition of oligomycin (0.5 µM, #495 455, Sigma Aldrich, St. Louis, USA). Following stabilization of respiration, FCCP (1 µM, #C2920, Sigma‐Aldrich, USA) was titrated in three manual increments to elicit maximal respiratory capacity. Finally, to measure non‐mitochondrial respiration, rotenone (Rot) and antimycin A (Ama)were administered. This step provides a measure of the residual oxygen consumption that is not linked to mitochondrial respiration.

### FAO Level Analysis

FAO enzyme activities were quantitatively assessed using fatty acid oxidation detection reagent (#FDV‐0033, Funakoshi, Japan). Cells were incubated with HBS containing the dissolved FAO Blue at 37 °C for 45 minutes to allow for adequate staining. Following incubation, cells were stained with SYTO Green nuclear dye (#KFS147, Beijing Baiaolaibo Technology, China). The SYTO Green dye was diluted 1:1500 and applied to the cells for 30 minutes. To assess FAO activity, the cells were visualized under a fluorescence microscope (BX53, OLYMPUS, Japan).

### RNA Sequencing

The RNA sequencing libraries were then prepared and sequenced by Shanghai Origin‐Gene Biomedical Technology Co., Ltd. The P values for statistical significance of expression differences were calculated using edgeR (v3.24), and adjustments for multiple testing were made to determine the false discovery rate (FDR). The results are shown in Table S1 (Supporting Information).

### PET28a Asprosin Prokaryotic Expression and Protein Purification

The asprosin gene was cloned into the PET28a vector and transformed into Rosetta (DE3) competent cells for enhanced expression of eukaryotic proteins. Initially, a small‐scale inducible expression test was conducted to confirm the expression of the asprosin protein. The cultures were induced and samples were analyzed using SDS‐PAGE to confirm the expression of a protein with an expected molecular weight of approximately 15 kDa. Following successful confirmation of protein expression, the culture was scaled up and the protein was purified. The asprosin protein was solubilized using 8 M urea. Subsequently, SDS‐PAGE was performed again to assess the purity and concentration of the purified protein. This step ensures that the protein is of sufficient quality and purity for further biochemical and structural analyses.

### His pull‐down and LC‐MS/MS Mass Spectrometry Identification

His pull‐down experiments were performed using the PolyHis Protein Interaction Pull‐Down Kit (#21 277, Thermo Fisher Scientific, USA). Initially, each column tube was filled with 50 uL of HisPur Cobalt Resin and washed five times by centrifugation at 1250 g for 1 min. The columns were then set aside. For the binding process, 400 uL of purified bait protein was added to each column and incubated at 4 °C for 1 h. Post‐incubation, collect the flow‐through, which was labeled as Bait flow‐through and stored at 4 °C. The bait‐decoy protein complex was then washed five times to ensure complete immobilization of the bait protein onto the packing. Total protein was extracted from HepG2 cells and added to the columns. The asprosin+ HepG2 group columns were incubated with gentle shaking at 4 °C for 2 h to enhance protein binding. The columns were then centrifuged at 1250 g for 1 minute to collect the column‐penetrating solution, which was stored at 4 °C for reuse. The protein complexes captured by the bait protein were eluted with 10 mM glutathione eluent, and the eluates were collected by centrifugation at 1250 g for 1 min. Gels were cut and prepared for mass spectrometry to identify interacting proteins. LC‐MS/MS analysis was managed by Shanghai Origin‐Gene Biomedical Technology Co., Ltd. The results are shown in Table S2 (Supporting Information).

### Quantification and Statistical Analysis

All data were expressed as mean ± SEM. Statistical analyses were performed using GraphPad Prism version 8.0. Differences between the two groups were assessed using Student's t‐test. For analyses involving more than two groups, one‐way ANOVA or two‐way ANOVA was applied, depending on the experimental design, followed by post‐hoc comparisons using Dunnett's or Tukey's multiple comparison tests. Randomized block ANOVA for data sets normalized to a control value of 1 and without SEM (repeated measures ANOVA). P‐value of less than 0.05 was considered to indicate statistical significance.

## Conflict of Interest

The authors declare no conflict of interest.

## Author Contributions

Y.Y.Y., M.F., and Y.Z. conceived and designed the study. Y.Y.Y., M.F., H.L.J., Q.Z., and Y.Z. underwent background checks. Y.X.L., J.L.S., and Y.Y.Y. helped analyse the data. Y.Y.Y., M.F., Y.C., Y.X.L., H.L.J., Q.Z., Y.Z., X.X.L., M.T., S.F.C., Y.Z., and Z.K.W. contributed to the statistics and graphing of the data. M.F., Y.C., Y.X.L., X.L., H.W.L., H.L.J., Q.Z., Y.Z., X.X.L., M.T., S.F.C., Y.Z., and Z.K.W. contributed to checking and revising the paper. Y.Y.Y. wrote the draft of the paper. Y.Y.Y., Y.Z., and Y.C. have completed data verification. All authors have thoroughly reviewed all the underlying data in the study, and confirmed it as the final version of the manuscript.

## Supporting information



Supporting Information

## Data Availability

The data that support the findings of this study are available from the corresponding author upon reasonable request.
