# Supplementary material for: Asprosin‐FABP5 Interaction Modulates Mitochondrial Fatty Acid Oxidation through PPARα Contributing to MASLD Development
Source: Adv Sci (Weinh). 2025 Apr 15;12(21):2415846. doi: 10.1002/advs.202415846 (PMC12140288; doi:10.1002/advs.202415846)
Supplement: Supplementary file 1 — Supporting Information [file ADVS-12-2415846-s001.docx]

## Asprosin-FABP5 Interaction Modulates Mitochondrial Fatty Acid Oxidation Through PPARα Contributing To MASLD Development

Yuan-Yuan Yu^1^, Min Feng^1^, Yi Chen^1^, Hong-Lin Jia^1^, Qi Zhang^1^, Ming Tong^1^, Yan-Xi Li^1^, Yu Zhao^1,2^, Xin-Xin Liu^1^, Shi-Feng Cao^1^, Zheng-kai Wang^1^, Hou-wei Li^3^, Xue Liu^1^, Yan Zhang^1, 4,^ *zhangyan@ems.hrbmu.edu.cn

^1^Department of Pharmacology, SKLFZCD, (State Key Labratoray -Province Key Laboratories of Biomedicine-Pharmaceutics of China, Key Laboratory of Cardiovascular Research, Ministry of Education), College of Pharmacy, Harbin Medical University, Harbin 150081, China

^2^Department of Pathophysiology (Province Key Laboratory of Medicine-Food Homologous Resources and Prevention and Treatment of Metabolic Diseases), Basic Medical College, Qiqihar Medical University, Qiqihar, 161000, China

^3^Department of Cardiology at the Second Affiliated Hospital of Harbin Medical University, SKLFZCD, Harbin, China

^4^Department of Pharmacology, State Key Laboratory of Frigid Zone Cardiovascular Diseases (SKLFZCD), Department of Pharmacy of The Second Affiliated Hospital, Harbin Medical University, Harbin 150081, China

Email: [zhangyan@ems.hrbmu.edu.cn](mailto:zhangyan@ems.hrbmu.edu.cn))

Department of Pharmacology,

Harbin Medical University,

157th Rd of Baojian, Nangang Distinct, Heilongjiang, China.

**Caption for supplementary material**

**Supplementary Figure S1.**

**Supplementary Figure S2.**

**Supplementary Figure S3.**

**Supplementary Figure S4.**

**Supplementary Figure S5.**

**Supplementary Figure S6.**

**Supplementary Figure S7.**

**Supplementary Table S1.** RNA sequencing

**Supplementary Table S2** His pull-down and LC-MS/MS mass spectrometry identification

**Supplementary Table S3** Primer sequences used in the study are shown as follows


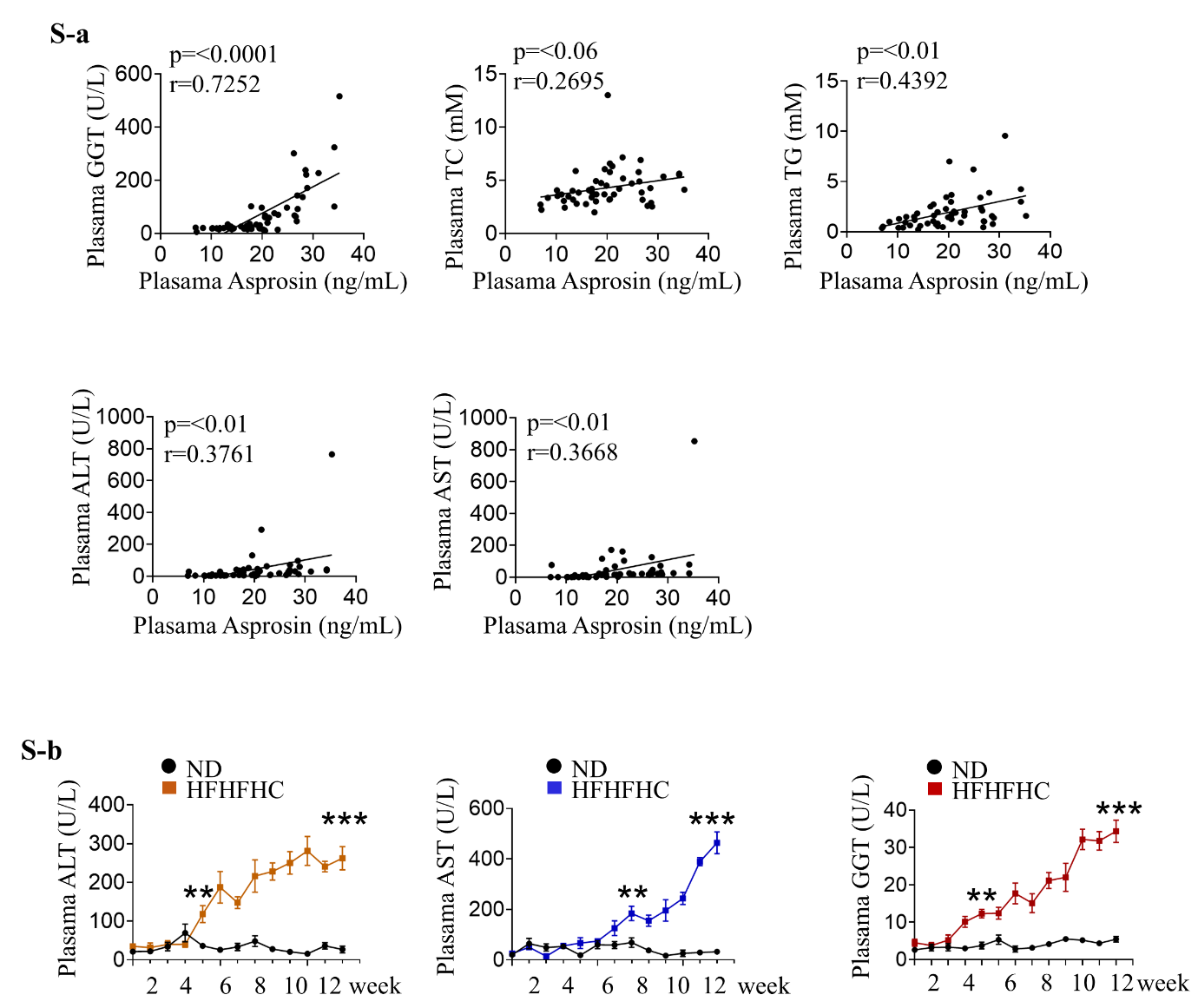
***Fig. 1:* Asprosin level increases in MASLD patients and experiment animals** (a) Correlation between plasma asprosin levels and changes in GGT, TC, TG, ALT, AST levels in MASLD patients and healthy control. n=52 in each group. (b) In the high-fat, high-cholesterol, high-fructose diet model, weekly detection of changes in serum aspartate aminotransferase (ALT), alanine aminotransferase (AST) and gamma-glutamyl transferase (GGT) levels in mice. n=6 in each group. HFHFHC, high-fat, high-cholesterol, high-fructose; ALT, aspartate aminotransferase; AST, alanine aminotransferase. Statistical analysis was performed with one-way ANOVA. ** *P* < 0.01, *** *P* < 0.001 vs NC.

***
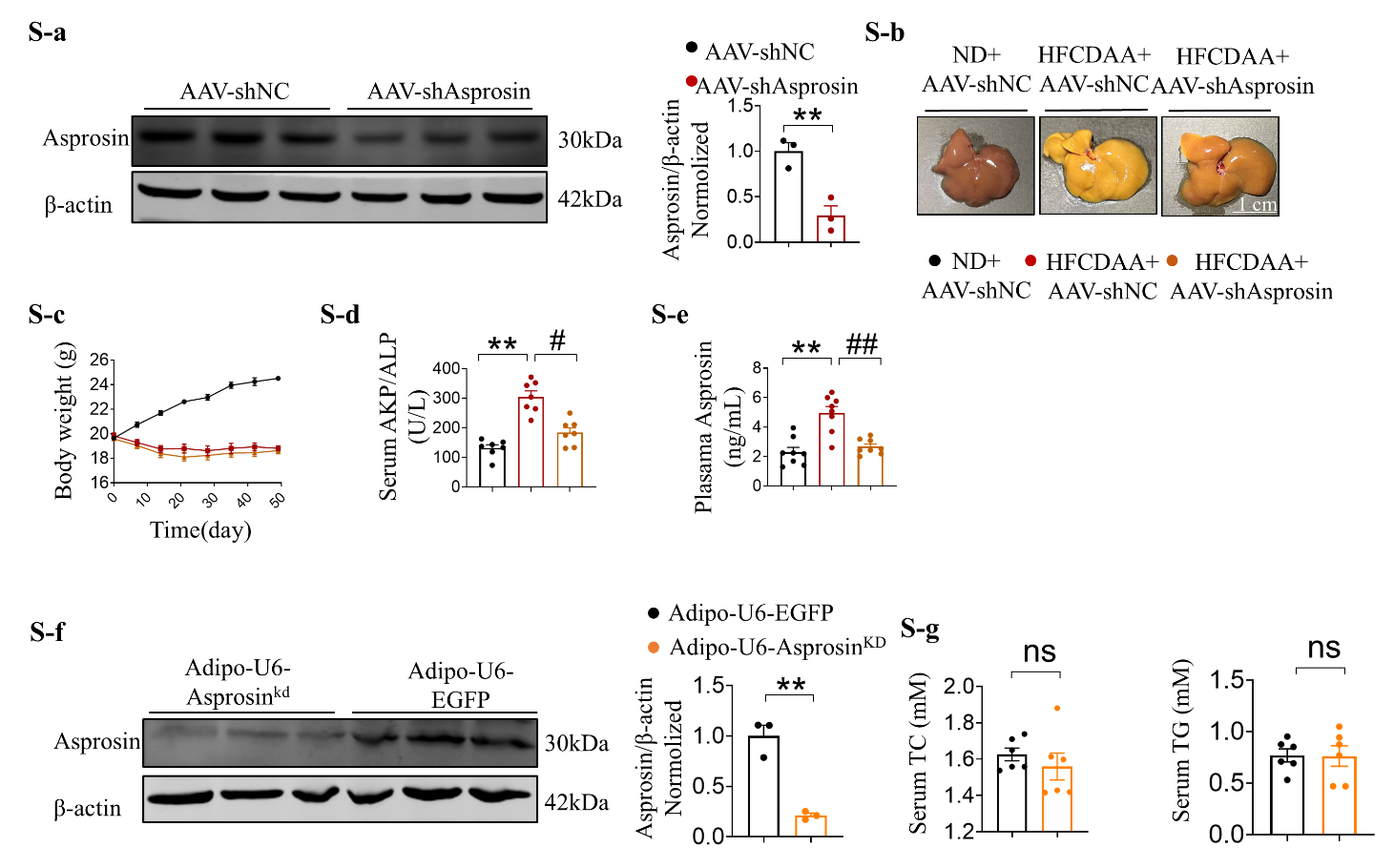
Fig. 2:* Hepatic asprosin deficiency alleviates hepatic steatosis.** (a) Western blot detection of the efficiency of asprosin protein knockdown in the liver eight weeks after the injection of AAV8-shAsprosin. n=3 in each group. (b) Representative images of livers from C57BL/6J mice fed with a high fat, methionine choline deficiency (HFCDAA) diet showing visible steatosis as evidenced by swollen and yellowed livers. n=8 in each group. (c) Body weight of C57BL/6J mice from different groups. n=8 in each group. (d) AAV-shAsprosin reduced the elevated serum transaminase level induced by HFCDAA feeding in C57BL/6J mice. AKP, alkaline phosphatase. n=6-8 in each group. (e) Plasma levels of asprosin in mice. n=8 in each group. (f) Western blot detection of the efficiency of asprosin protein knockdown in the adipose tissue eight weeks after the injection of AAV8-shAsprosin. n=3 in each group. (g) The serum TC, TG levels. n=6 in each group. Statistical analysis was performed with one-way ANOVA. * *P* < 0.05, ** *P* < 0.01 vs ND+AAV-shNC; # *P* < 0.05, ## *P* < 0.01 vs HFCDAA+AAV-NC; ns, no significanse.

***
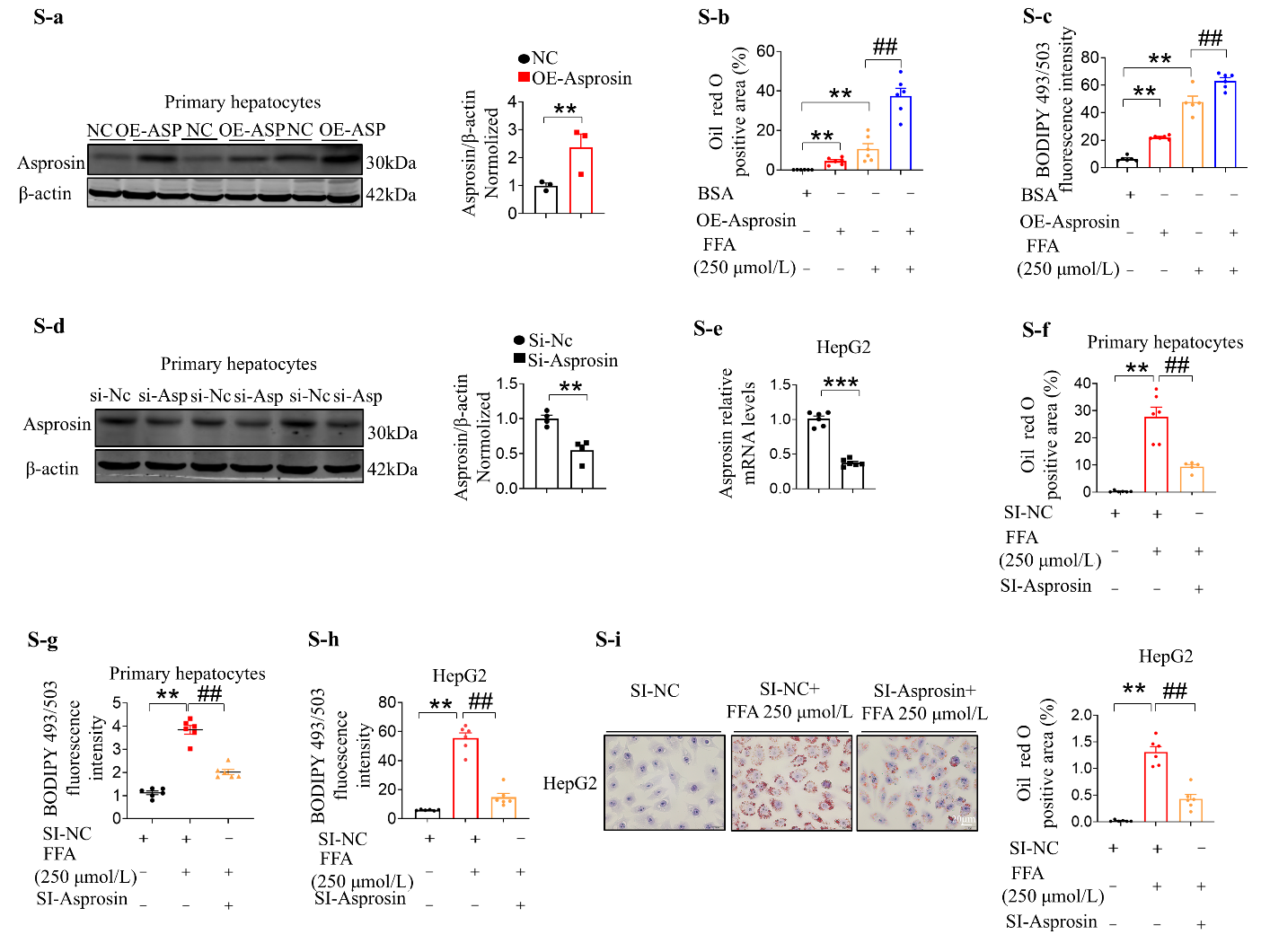
Fig. 3:* Asposin induces lipid accumulation in HepG2 cells and primary hepatocytes.** (a) Western blot detection of overexpressed asprosin protein levels in primary hepatocytes. n=3 in each group. (b-c) Statistical of Oil Red O and BODIPY staining in primary hepatocytes. Cells were transfected with an asprosin overexpression plasmid for 24 hours, followed by stimulation with 250 µM free fatty acids (FFA) for another 24 hours. n=5, 6 in each group. (d) Western blot detection of knockdown asprosin protein levels in primary hepatocytes. n=4 in each group. (e) qPCR detection of knockdown asprosin mRNA levels in HepG2 cells. n=6 in each group. (f-g) Statistical of Oil Red O and BODIPY staining in primary hepatocytes. n=6 in each group. ((h-i) Statistical of Oil Red O and BODIPY staining in HepG2 cells. n=6 in each group. ASP, asprosin. Scale bar for Oil red O staining: 20 μm, for BODIPY staining: 10 μm. Statistical analysis was performed with one-way ANOVA. * *P* < 0.05, ** *P* < 0.01, *** *P* < 0.001 vs NC; ## *P* < 0.01 vs FFA (250 μM).

***
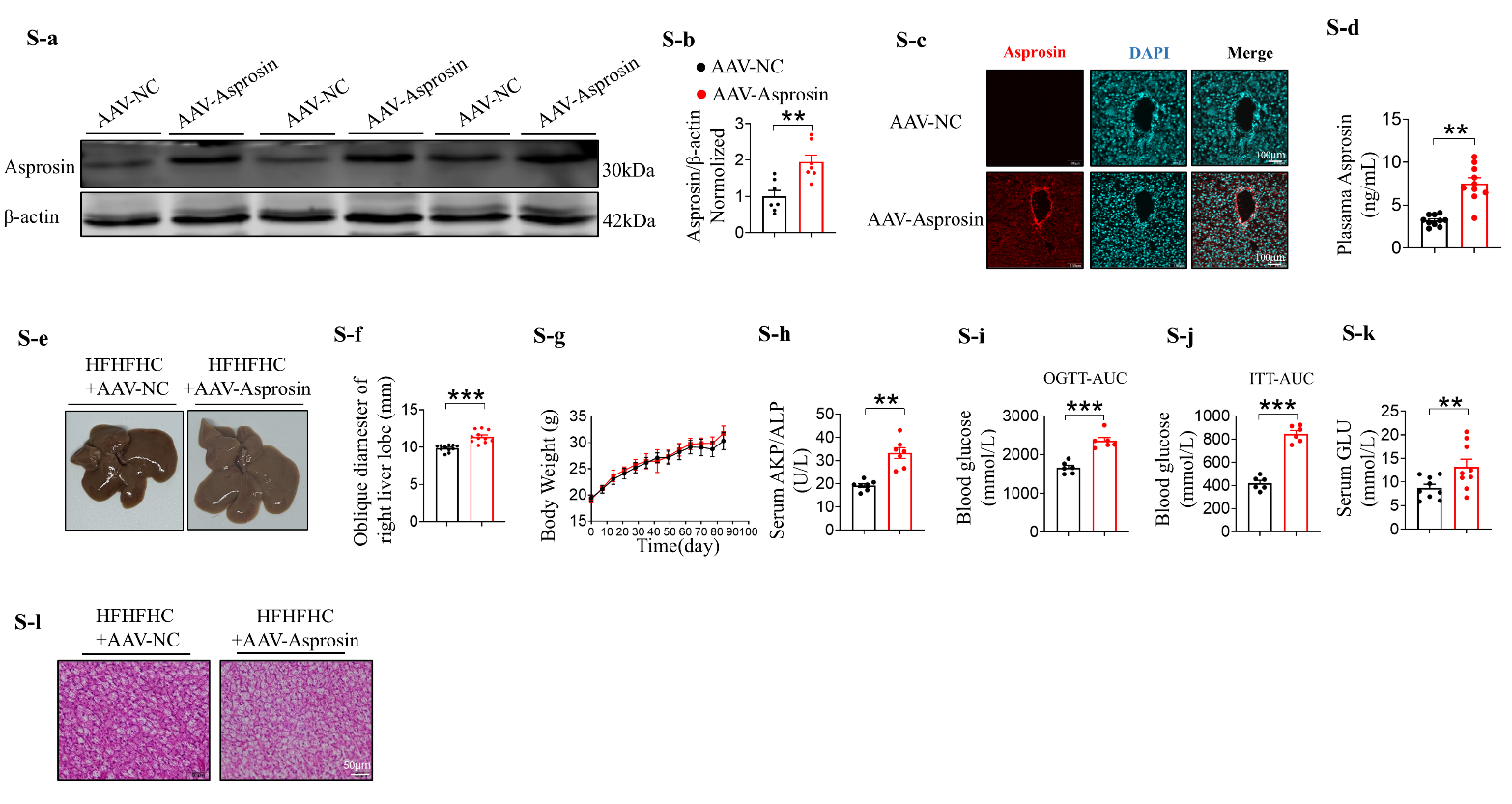
Fig. 4:* Asprosin exacerbates HFHFHC diet-induced hepatic steatosis, hyperlipidaemia and increases insulin resistance in APOE^(-/-)^ mice.** (a-c) Endogenous asprosin protein changes in the liver detected by Western blot and immunofluorescence after hepatic overexpression of asprosin. n=6 in each group. (d) Plasma levels of asprosin in mice. n=9-10 in each group. (e) Representative images of livers from each experimental group. n=12 in each group. (f) Statistical analysis of the oblique diameter of the right lobe of the liver as determined by Doppler ultrasound. n=11 in each group. (g) Analyzed data for body weight. n=10-12 in each group. (h, k) Analyzed data of serum AKP and GLU levels in APOE^(-/-)^ mice. n=7-10 in each group. (i-j) Area under the OGTT and ITT curves statistics. n=6 in each group. (l) HE staining of brown fat. n=6 in each group. Scale bar for fluorescence staining:100 μm; for HE staining:50 μm. Statistical analysis was performed with one-way ANOVA. * *P* < 0.05, ** *P* < 0.01, *** *P* < 0.001 vs HFHFHC+AAV-NC.

***
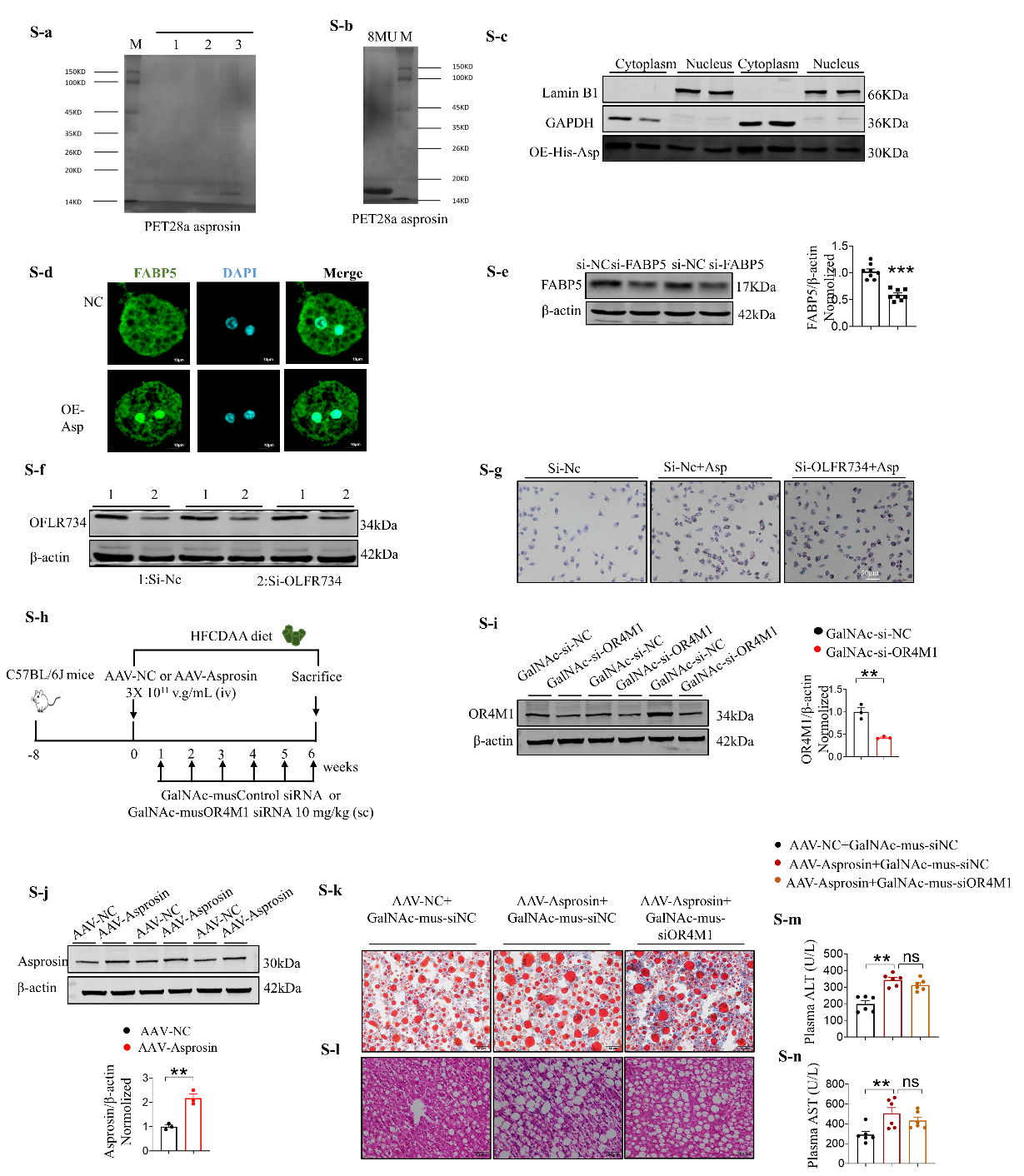
Fig. 5:* Asprosin-FABP5 interaction and nuclear translocation in hepatic cells.** (a) Western blot detection of induced expression of PET28a-asprosin protein. (b) Western blot detectionof PET28a-asprosin protein purification. (c) Following the overexpression of the asprosin plasmid in HepG2 cells for 48 hours, cytoplasmic and nuclear protein isolation assays were conducted. The distribution of asprosin in the cells was detected by Western blot. n =3 in each group. (d) Immunofluorescence shows that asprosin promotes FABP5 nuclear translocation. Primary hepatocytes were transfected with His-asprosin before immunofluorescence staining. FABP5 (green), DAPI (blue). n =4, 6 in each group. (e) Western blot assay for knockdown of FABP5 efficiency in HepG2 cells. n=3-8 in each group. (f) Western blot assay for knockdown of OLFR734 efficiency in HepG2 cells. n =8 in each group. (g) Oil red O staining to detect the effect of knockdown of OLFR734 on asprosin-induced lipid accumulation. n =6 in each group. (h) Schematic illustration of the experiment design, with an overview of GalNAc-siOR4M1 injection time points. (i) Western blot assay for knockdown of OR4M1 efficiency in livers. n=3 in each group. (j) Endogenous asprosin protein changes in the liver detected by Western blot after hepatic overexpression of asprosin. n=3 in each group. (k-l) Representative histological images of liver biopsies stained with Oil Red O and H&E staining. n=6 in each group. (m-n). Serum ALT and AST levels in C57BL/6J mice. n=6 in each group.

Scale bar for FABP5 immunofluorescence: 10 μm; for Oil red O staining: 50 μm. Statistical analysis was performed with one-way ANOVA. *** *P* < 0.001 vs NC; ns, no significanse.

***
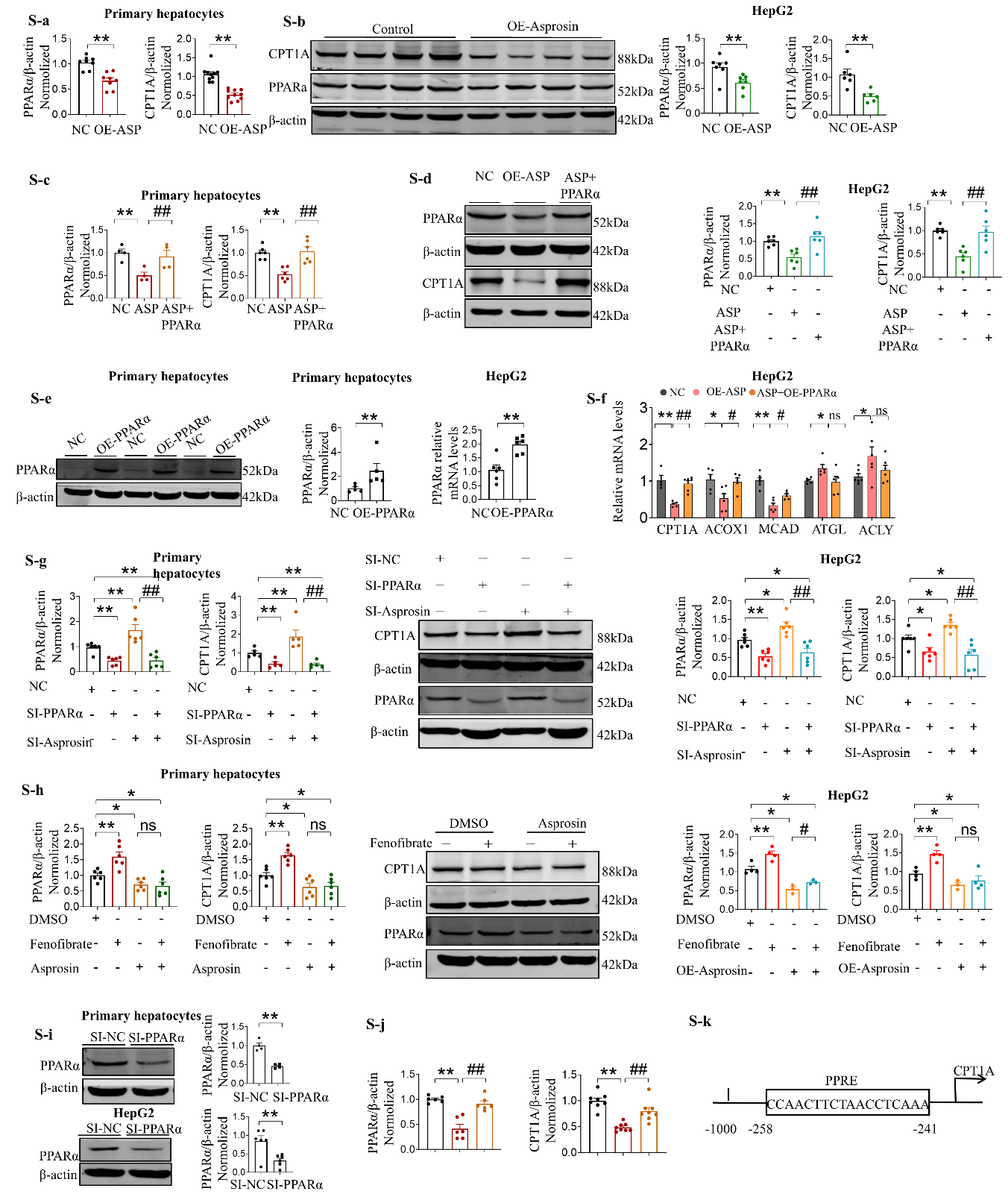
Fig. 6:* Asprosin targets the PPRE of PPARα to modulate CPT1A expression and mitochondrial function.** (a) Statistics of asprosin overexpression effects on PPARα and CPT1A protein expression. n=8 in each group. ** *P* < 0.01 vs NC. (b) Asprosin inhibited the expression of PPARα and its target gene CPT1A in HepG2 cells. n=6, 7 in each group. ** *P* < 0.01 vs control. (c-d) Asprosin reduced the expression and activity of PPARα, but cotransfected PPARα expression vector restored them. n=6 in each group. ** *P* < 0.01 vs NC; ## *P* < 0.01 vs Asp. (e) Western blot assay for overexpression of PPARα efficiency in primary hepatocytes. n=5 in each group. ** *P* < 0.01 vs NC. (f) qPCR to detect the effect of PPARα overexpression on mRNA of lipid metabolism genes related to asprosin effects. n=4-6 in each group. ** *P* < 0.01 vs NC; ## *P* < 0.01 vs Asp. (g) Statistics of PPARα and CPT1A protein expression in primary hepatocytes; PPARα- targeting siRNA (siPPARα) suppressed the expression of PPARα. The upregulation of genes by si-Asprosin was also suppressed by siPPARα HepG2 cells transfected with indicated siRNA, or PPARα expression vector. Western blot analysis of PPARα and CPT1A. n=6 in each group. * *P* < 0.05, ** *P* < 0.01 vs NC; # *P* < 0.05 vs SI-Asprosin. (h) Statistics of PPARα and CPT1A protein expression in primary hepatocytes. n=3-6 in each group. * *P* < 0.05 , ** *P* < 0.01 vs DMSO; # *P* < 0.05 vs Asp; ns, not significant. (i) Western blot for knockdown efficiency of PPARα protein in HepG2 cells and primary hepatocytes. n=6 in each group.** *P* < 0.01 vs NC. (j) Statistics of PPARα and CPT1A protein expression in HepG2 cells. n=4-8 in each group. (k) The binding site of PPARα protein in CPT1A was predicted by JASPAR database, which has the highest coefficient and contains PPRE sequence, and primers were designed on both sides of the predicted binding site to amplify the predicted site to. Statistical analysis was performed with one-way ANOVA. ** *P* < 0.01 vs NC; ## *P* < 0.01 vs Asprosin.

***
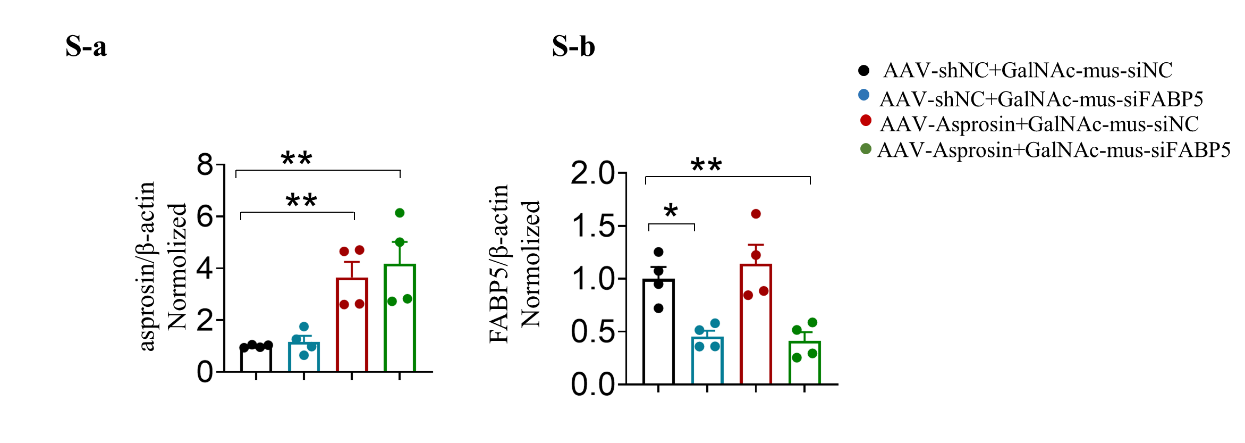
Fig. 7:* AAV-shAsprosin enhances effects of fenofibrate in HFCDAA-fed mice.** (a-b) Statistics of asprosin and FABP5 protein expression. * *P* < 0.05, ** *P* < 0.01 VS AAV-shNC+GalNAc-mus-siNC.

**Supplementary Tables**

**Table S1 RNA sequencing**

| **Down**  **TermID** | **Description** | **P_value** | **GeneNames** |
| --- | --- | --- | --- |
| ko03320 | PPAR signaling pathway | 2.82E-10 | Acaa1a,Acaa1b,Acadl,Acadm,Acox1,Angptl4,Apoa2,Apoc3,Cpt1a,Cpt2,Cyp4a10,Cyp4a31,Cyp4a32,Cyp7a1,Cyp8b1,Ehhadh,Fabp1,Fabp7,Fads2,Gk,Plin2,Ppara,Scp2-ps2,Slc27a1,Slc27a2,Slc27a5 |
| ko04932 | Non-alcoholic fatty liver disease (NAFLD) | 4.31E-08 | Adipor1,Bid,Cebpa,Cox4i1,Cox5a,Cox5b,Cox6a1,Cox6b1,Cox7b,Cox7c,Cox8a,Cyp2e1,Gm12338,Gsk3a,Ndufa11,Ndufa13,Ndufb10,Ndufb6,Ndufb8,Ndufb9,Ndufs2,Ndufs4,Ndufs6,Ppara,Sdhb,Sdhd,Uqcr10,Uqcr11,Uqcrc1,Uqcrfs1,Uqcrq |
| ko01212 | Fatty acid metabolism | 6.08E-07 | Acaa1a,Acaa1b,Acaa2,Acadl,Acadm,Acadvl,Acox1,Cpt1a,Cpt2,Ehhadh,Fads2,Hacd3,Hadh,Hadha,Hadhb,Hadhb-ps |
| ko00770 | Pantothenate and CoA biosynthesis | 1.75E-05 | Bcat2,Coasy,Dpys,Pank1,Ppcs,Upb1,Vnn1,Vnn3 |
| ko00120 | Primary bile acid biosynthesis | 1.89E-05 | Acot8,Akr1d1,Amacr,Cyp7a1,Cyp8b1,Hsd17b4,Scp2-ps2,Slc27a5 |
| ko00330 | Arginine and proline metabolism | 2.50E-05 | Agmat,Aldh2,Aldh7a1,Aldh9a1,Azin2,Gamt,Gm21969,Got2,Hoga1,L3hypdh,Prodh,Prodh2 |
| ko00410 | beta-Alanine metabolism | 9.30E-05 | Acadm,Aldh2,Aldh7a1,Aldh9a1,Dpys,Ehhadh,Hadha,Mlycd,Upb1 |
| ko00640 | Propanoate metabolism | 0.000134427 | Acadm,Acss3,Bckdha,Bckdhb,Ehhadh,Hadha,Mcee,Mlycd,Suclg1 |
| ko00983 | Drug metabolism - other enzymes | 0.000135874 | Cda,Ces1d,Ces1e,Ces2e,Dpys,Tk1,Uck1,Ugt1a2,Ugt1a6b,Ugt2a3,Ugt2b36,Upb1 |
| ko00650 | Butanoate metabolism | 0.000186204 | Acsm3,Bdh1,Ehhadh,Hadh,Hadha,Hmgcl,Hmgcs2 |
| ko01230 | Biosynthesis of amino acids | 0.000351424 | Acy1,Aldh7a1,Aldoc,Asl,Bcat2,Eno3,Gm10293,Gm3839,Got2,Gpt,Gpt2,Mat1a,Mat2a,Mat2b,Shmt1,Tha1 |

| **Up**  **TermID** | **Description** | **P_value** | **GeneNames** |
| --- | --- | --- | --- |
| ko04710 | Circadian rhythm | 0.00532372 | Cry1,Npas2 |
| ko04740 | Olfactory transduction | 0.007506926 | Arrb1,Olfr1033 |
| ko05130 | Pathogenic Escherichia coli infection | 0.010709522 | Tuba4a,Tubb2a |
| ko00520 | Amino sugar and nucleotide sugar metabolism | 0.014412776 | Gale,Gmds |
| ko04540 | Gap junction | 0.037170826 | Tuba4a,Tubb2a |
| ko05032 | Morphine addiction | 0.044466114 | Arrb1,Pde4b |

**Table S2 His pull-down and LC-MS/MS mass spectrometry identification**

| **Accession** | **Gene names** | **Exp. q-value: Combined** | **Protein FDR Confidence: Combined** |
| --- | --- | --- | --- |
| P02768 | ALB | 0 | High |
| O43143 | DHX15 | 0 | High |
| P04264 | KRT1 | 0 | High |
| P35527 | KRT9 | 0 | High |
| Q9HC35 | EML4 | 0 | High |
| P68104 | EEF1A1 | 0 | High |
| Q9H6R3 | ACSS3 | 0 | High |
| P35908 | KRT2 | 0 | High |
| P04259 | KRT6B | 0 | High |
| P02538 | KRT6A | 0 | High |
| P48668 | KRT6C | 0 | High |
| O95479 | H6PD | 0 | High |
| P51659 | HSD17B4 | 0 | High |
| P49748 | ACADVL | 0 | High |
| A0A1B0GVI3 | KRT10 | 0 | High |
| Q86XP3 | DDX42 | 0 | High |
| P29401 | TKT | 0 | High |
| P13639 | EEF2 | 0 | High |
| Q9Y6N5 | SQOR | 0 | High |
| Q15436 | SEC23A | 0 | High |
| P08779 | KRT16 | 0 | High |
| Q9ULV4 | CORO1C | 0 | High |
| P13647 | KRT5 | 0 | High |
| P11142 | HSPA8 | 0 | High |
| O75083 | WDR1 | 0 | High |
| P02533 | KRT14 | 0 | High |
| P23921 | RRM1 | 0 | High |
| P00367 | GLUD1 | 0 | High |
| P55072 | VCP | 0 | High |
| D6REX3 | SEC31A | 0 | High |
| P02787 | TF | 0 | High |
| A0A2R8Y5A3 | CTNNB1 | 0 | High |
| P34897 | SHMT2 | 0 | High |
| A0A494C0R8 | CLUH | 0 | High |
| Q8WWM7 | ATXN2L | 0 | High |
| P05787 | KRT8 | 0 | High |
| Q86TI2 | DPP9 | 0 | High |
| F5GXS2 | ACTN4 | 0 | High |
| P10809 | HSPD1 | 0 | High |
| H3BUF6 | ATXN2L | 0 | High |
| P15924 | DSP | 0 | High |
| P11021 | HSPA5 | 0 | High |
| O94855 | SEC24D | 0 | High |
| P0C0L5 | C4B_2 | 0 | High |
| Q15459 | SF3A1 | 0 | High |
| Q96RS6 | NUDCD1 | 0 | High |
| P53992 | SEC24C | 0 | High |
| P49327 | FASN | 0 | High |
| P12931 | SRC | 0 | High |
| Q99536 | VAT1 | 0 | High |
| P35606 | COPB2 | 0 | High |
| Q04695 | KRT17 | 0 | High |
| Q9C0I1 | MTMR12 | 0 | High |
| A0A2U3TZH3 | EEF1A2 | 0 | High |
| P23368 | ME2 | 0 | High |
| O14745 | SLC9A3R1 | 0 | High |
| O60701 | UGDH | 0 | High |
| P21333 | FLNA | 0 | High |
| Q86YZ3 | HRNR | 0 | High |
| Q2TAY7 | SMU1 | 0 | High |
| P25205 | MCM3 | 0 | High |
| P14923 | JUP | 0 | High |
| P28482 | MAPK1 | 0 | High |
| Q14247 | CTTN | 0 | High |
| I3L504 | EIF5A | 0 | High |
| A0A7P0T9U7 | SF1 | 0 | High |
| Q8WUA2 | PPIL4 | 0 | High |
| A0A7P0Z472 | ADSL | 0 | High |
| C9JC84 | FGG | 0 | High |
| P04406 | GAPDH | 0 | High |
| P14866 | HNRNPL | 0 | High |
| A0A804HII9 | ACTN1 | 0 | High |
| E7EVA0 | MAP4 | 0 | High |
| P23786 | CPT2 | 0 | High |
| Q3LXA3 | TKFC | 0 | High |
| Q06830 | PRDX1 | 0 | High |
| P01023 | A2M | 0 | High |
| O43660 | PLRG1 | 0 | High |
| J3KQE5 | RAN | 0 | High |
| P34932 | HSPA4 | 0 | High |
| Q9HA77 | CARS2 | 0 | High |
| P53396 | ACLY | 0 | High |
| O95817 | BAG3 | 0 | High |
| Q15437 | SEC23B | 0 | High |
| Q9NVX2 | NLE1 | 0 | High |
| H0YAB3 | SEC31A | 0 | High |
| Q53GS9 | USP39 | 0 | High |
| A0A7I2V535 | NONO | 0 | High |
| P50395 | GDI2 | 0 | High |
| J3QR07 | YTHDC1 | 0 | High |
| P07099 | EPHX1 | 0 | High |
| Q15393 | SF3B3 | 0 | High |
| Q7Z5L9 | IRF2BP2 | 0 | High |
| Q9P0Z9 | PIPOX | 0 | High |
| P11940 | PABPC1 | 0 | High |
| A0A8I5KZ76 | MTM1 | 0 | High |
| O95425 | SVIL | 0 | High |
| Q96EE3 | SEH1L | 0 | High |
| P22681 | CBL | 0 | High |
| P50552 | VASP | 0 | High |
| Q9NUQ6 | SPATS2L | 0 | High |
| Q9GZV4 | EIF5A2 | 0 | High |
| P60709 | ACTB | 0 | High |
| C9JA08 | NMD3 | 0 | High |
| Q9UHN6 | CEMIP2 | 0 | High |
| A0A6Q8PFJ0 | LMNA | 0 | High |
| O43813 | LANCL1 | 0 | High |
| Q8N163 | CCAR2 | 0 | High |
| J3KPS3 | ALDOA | 0 | High |
| Q9NW82 | WDR70 | 0 | High |
| P13646 | KRT13 | 0 | High |
| O15042 | U2SURP | 0 | High |
| P21127 | CDK11B | 0 | High |
| A0A0A0MS08 | IGHG1 | 0 | High |
| P14618 | PKM | 0 | High |
| Q96QZ7 | MAGI1 | 0 | High |
| Q9BTU6 | PI4K2A | 0 | High |
| P33992 | MCM5 | 0 | High |
| Q16706 | MAN2A1 | 0 | High |
| Q96HC4 | PDLIM5 | 0 | High |
| P40818 | USP8 | 0 | High |
| P61626 | LYZ | 0 | High |
| P30533 | LRPAP1 | 0 | High |
| Q3BDU5 | LMNA | 0 | High |
| A0A669KBL1 | USP7 | 0 | High |
| Q9NUW8 | TDP1 | 0 | High |
| P07900 | HSP90AA1 | 0 | High |
| Q92900 | UPF1 | 0 | High |
| P45880 | VDAC2 | 0 | High |
| Q9GZT9 | EGLN1 | 0 | High |
| Q15067 | ACOX1 | 0 | High |
| P06733 | ENO1 | 0 | High |
| Q04724 | TLE1 | 0 | High |
| Q9UK59 | DBR1 | 0 | High |
| P32322 | PYCR1 | 0 | High |
| Q9Y2W1 | THRAP3 | 0 | High |
| Q9HCC0 | MCCC2 | 0 | High |
| P41222 | PTGDS | 0 | High |
| Q9NQC3 | RTN4 | 0 | High |
| P23246 | SFPQ | 0 | High |
| Q16531 | DDB1 | 0 | High |
| Q92896 | GLG1 | 0 | High |
| A0A5F9ZHN9 | ALDH3A2 | 0 | High |
| P29966 | MARCKS | 0 | High |
| Q15084 | PDIA6 | 0 | High |
| Q16630 | CPSF6 | 0 | High |
| P26440 | IVD | 0 | High |
| Q02252 | ALDH6A1 | 0 | High |
| P00352 | ALDH1A1 | 0 | High |
| Q02413 | DSG1 | 0 | High |
| P25098 | GRK2 | 0 | High |
| P05783 | KRT18 | 0 | High |
| P38646 | HSPA9 | 0 | High |
| P02649 | APOE | 0 | High |
| P19013 | KRT4 | 0 | High |
| Q04727 | TLE4 | 0 | High |
| P68871 | HBB | 0 | High |
| Q16204 | CCDC6 | 0 | High |
| E9PAU2 | RAVER1 | 0 | High |
| P22059 | OSBP | 0 | High |
| P78371 | CCT2 | 0 | High |
| Q9GZP4 | PITHD1 | 0 | High |
| A0A5F9UY30 | TGOLN2 | 0 | High |
| P08727 | KRT19 | 0 | High |
| Q16658 | FSCN1 | 0 | High |
| Q9BR76 | CORO1B | 0 | High |
| P27361 | MAPK3 | 0 | High |
| Q8N1G2 | CMTR1 | 0 | High |
| A0A590UJ43 | CSNK1A1 | 0 | High |
| O95081 | AGFG2 | 0 | High |
| A0A7P0TAE9 | CANX | 0 | High |
| H7BY16 | NCL | 0 | High |
| P48634 | PRRC2A | 0 | High |
| P69905 | HBA2 | 0 | High |
| Q99700 | ATXN2 | 0 | High |
| Q13435 | SF3B2 | 0 | High |
| Q9Y520 | PRRC2C | 0 | High |
| Q5MNZ6 | WDR45B | 0 | High |
| O60664 | PLIN3 | 0 | High |
| Q86Y46 | KRT73 | 0 | High |
| Q00796 | SORD | 0 | High |
| O14672 | ADAM10 | 0 | High |
| P23526 | AHCY | 0 | High |
| Q9BSH4 | TACO1 | 0 | High |
| Q9UHD8 | SEPTIN9 | 0 | High |
| Q9BZK7 | TBL1XR1 | 0 | High |
| E9PHY5 | EPB41L2 | 0 | High |
| P0DOY2 | IGLC2 | 0 | High |
| Q9P0L0 | VAPA | 0 | High |
| Q13200 | PSMD2 | 0 | High |
| Q8WXF1 | PSPC1 | 0 | High |
| P32119 | PRDX2 | 0 | High |
| Q06323 | PSME1 | 0 | High |
| A0A0G2JIW1 | HSPA1B | 0 | High |
| Q9P2E9 | RRBP1 | 0 | High |
| Q13310 | PABPC4 | 0 | High |
| A0A2R8YDH4 |  | 0 | High |
| Q08752 | PPID | 0 | High |
| Q15785 | TOMM34 | 0 | High |
| Q9NYF8 | BCLAF1 | 0 | High |
| J3QRU1 | YES1 | 0 | High |
| A0A7I2V4B3 | ADAM9 | 0 | High |
| G3V1L9 | TJP1 | 0 | High |
| A0A7P0TA71 | P4HB | 0 | High |
| Q92835 | INPP5D | 0 | High |
| O75369 | FLNB | 0 | High |
| H0YFD6 | HADHA | 0 | High |
| P61158 | ACTR3 | 0 | High |
| Q8N1N4 | KRT78 | 0 | High |
| P31689 | DNAJA1 | 0 | High |
| P38606 | ATP6V1A | 0 | High |
| P15144 | ANPEP | 0 | High |
| Q92979 | EMG1 | 0 | High |
| Q8WVV9 | HNRNPLL | 0 | High |
| Q8NBS9 | TXNDC5 | 0 | High |
| Q96EN8 | MOCOS | 0 | High |
| Q13724 | MOGS | 0 | High |
| Q9GZN8 | C20orf27 | 0 | High |
| P19823 | ITIH2 | 0 | High |
| Q09666 | AHNAK | 0 | High |
| P08238 | HSP90AB1 | 0 | High |
| P55735 | SEC13 | 0 | High |
| P15848 | ARSB | 0 | High |
| P05362 | ICAM1 | 0 | High |
| P13674 | P4HA1 | 0 | High |
| P28799 | GRN | 0 | High |
| Q9HCE5 | METTL14 | 0 | High |
| Q7L2J0 | MEPCE | 0 | High |
| O43684 | BUB3 | 0 | High |
| Q8N6H7 | ARFGAP2 | 0 | High |
| P02671 | FGA | 0 | High |
| P01834 | IGKC | 0 | High |
| H7BYT1 | CSNK1D | 0 | High |
| A0A0B4J1R4 | HPD | 0 | High |
| Q5JPE7 | NOMO2 | 0 | High |
| P22234 | PAICS | 0 | High |
| Q7Z2K6 | ERMP1 | 0 | High |
| Q9ULJ6 | ZMIZ1 | 0 | High |
| Q15814 | TBCC | 0 | High |
| P78406 | RAE1 | 0 | High |
| Q5T6F2 | UBAP2 | 0 | High |
| Q5T440 | IBA57 | 0 | High |
| P62380 | TBPL1 | 0 | High |
| Q9UK58 | CCNL1 | 0 | High |
| Q8N684 | CPSF7 | 0 | High |
| O00468 | AGRN | 0 | High |
| Q16881 | TXNRD1 | 0 | High |
| A0A8I5KUL7 | FAM162A | 0 | High |
| H0YL70 | TLE3 | 0 | High |
| P35555 | FBN1 | 0 | High |
| P35221 | CTNNA1 | 0 | High |
| A0A7P0TAT8 | HSP90B1 | 0 | High |
| O95831 | AIFM1 | 0 | High |
| Q00839 | HNRNPU | 0 | High |
| A0A0A0MRM9 | NOLC1 | 0 | High |
| P50336 | PPOX | 0 | High |
| A0A3B3IS55 | ABLIM1 | 0 | High |
| Q9Y2S7 | POLDIP2 | 0 | High |
| Q9NS86 | LANCL2 | 0 | High |
| Q15691 | MAPRE1 | 0 | High |
| Q12797 | ASPH | 0 | High |
| Q15599 | SLC9A3R2 | 0 | High |
| P01034 | CST3 | 0 | High |
| O14744 | PRMT5 | 0 | High |
| Q14697 | GANAB | 0 | High |
| A0A804HI25 | DHCR7 | 0 | High |
| Q9Y5A9 | YTHDF2 | 0 | High |
| P33908 | MAN1A1 | 0 | High |
| Q9NSB2 | KRT84 | 0 | High |
| P01024 | C3 | 0 | High |
| A0A7I2V490 | DNMT1 | 0 | High |
| Q92636 | NSMAF | 0 | High |
| P05091 | ALDH2 | 0 | High |
| A0A3B3ISK9 | ACSF3 | 0 | High |
| Q9P258 | RCC2 | 0 | High |
| E9PK25 | CFL1 | 0 | High |
| O60869 | EDF1 | 0 | High |
| Q9UKX7 | NUP50 | 0 | High |
| P68036 | UBE2L3 | 0 | High |
| Q96I51 | RCC1L | 0 | High |
| Q6ZRP7 | QSOX2 | 0 | High |
| Q15075 | EEA1 | 0 | High |
| O75116 | ROCK2 | 0 | High |
| Q9HCU5 | PREB | 0 | High |
| Q9UHQ9 | CYB5R1 | 0 | High |
| Q9BWE0 | REPIN1 | 0 | High |
| A0A7P0TB19 | PPIL2 | 0 | High |
| O00267 | SUPT5H | 0 | High |
| Q9BVS5 | TRMT61B | 0 | High |
| P81605 | DCD | 0 | High |
| P25705 | ATP5F1A | 0 | High |
| O60907 | TBL1X | 0 | High |
| P15291 | B4GALT1 | 0 | High |
| A0A087X0X3 | HNRNPM | 0 | High |
| P15559 | NQO1 | 0 | High |
| O60885 | BRD4 | 0 | High |
| Q9Y320 | TMX2 | 0 | High |
| A0A286YEY1 | IGHA1 | 0 | High |
| Q15061 | WDR43 | 0 | High |
| Q9NYU2 | UGGT1 | 0 | High |
| Q9P265 | DIP2B | 0 | High |
| Q9H0D6 | XRN2 | 0 | High |
| A0A087WY31 | YTHDF3 | 0 | High |
| F6XFR5 | ABLIM1 | 0 | High |
| Q96F07 | CYFIP2 | 0 | High |
| A0A286YEY4 | IGHG2 | 0 | High |
| Q96C36 | PYCR2 | 0 | High |
| P43487 | RANBP1 | 0 | High |
| Q15424 | SAFB | 0 | High |
| P16402 | H1-3 | 0 | High |
| Q8NF64 | ZMIZ2 | 0 | High |
| J3QK89 | CHERP | 0 | High |
| Q8N5K1 | CISD2 | 0 | High |
| P53350 | PLK1 | 0 | High |
| A0A2R8Y5S7 | RDX | 0 | High |
| A0A0A0MTJ9 | NCEH1 | 0 | High |
| P07384 | CAPN1 | 0 | High |
| P22314 | UBA1 | 0 | High |
| D6RER5 | SEPTIN11 | 0 | High |
| Q92499 | DDX1 | 0 | High |
| P67809 | YBX1 | 0 | High |
| Q96H79 | ZC3HAV1L | 0 | High |
| Q9NVJ2 | ARL8B | 0 | High |
| E7EPK1 | SEPTIN7 | 0 | High |
| O60488 | ACSL4 | 0 | High |
| Q9Y2R0 | COA3 | 0 | High |
| Q15365 | PCBP1 | 0 | High |
| Q32Q12 | NME1-NME2 | 0 | High |
| Q6UXN9 | WDR82 | 0 | High |
| Q92598 | HSPH1 | 0 | High |
| O75376 | NCOR1 | 0 | High |
| A6XMV9 | PRSS2 | 0 | High |
| P00338 | LDHA | 0 | High |
| Q6P6C2 | ALKBH5 | 0 | High |
| C9J6P4 | ZC3HAV1 | 0 | High |
| A9Z1X7 | SRRM1 | 0 | High |
| A0A0A0MQR2 | RTF2 | 0 | High |
| Q15428 | SF3A2 | 0 | High |
| A0A0U1RRL8 | SPATA20 | 0 | High |
| Q9BQ52 | ELAC2 | 0 | High |
| P26368 | U2AF2 | 0 | High |
| P13051 | UNG | 0 | High |
| A0A0A6YYJ8 | LUC7L2 | 0 | High |
| A0A286YES1 | IGHG3 | 0 | High |
| A0A087WV05 |  | 0 | High |
| Q08426 | EHHADH | 0 | High |
| Q5T0F9 | CC2D1B | 0 | High |
| Q6PJ69 | TRIM65 | 0 | High |
| A0A1C7CYX9 | DPYSL2 | 0 | High |
| Q93099 | HGD | 0 | High |
| P15531 | NME1 | 0 | High |
| F6VRR5 | POLDIP3 | 0 | High |
| P31939 | ATIC | 0 | High |
| P15311 | EZR | 0 | High |
| P26641 | EEF1G | 0 | High |
| Q8IX18 | DHX40 | 0 | High |
| Q8NC51 | SERBP1 | 0 | High |
| P78314 | SH3BP2 | 0 | High |
| Q5T749 | KPRP | 0 | High |
| P04040 | CAT | 0 | High |
| P08621 | SNRNP70 | 0 | High |
| Q14151 | SAFB2 | 0 | High |
| Q9NP61 | ARFGAP3 | 0 | High |
| P21796 | VDAC1 | 0 | High |
| Q8NE62 | CHDH | 0 | High |
| Q15154 | PCM1 | 0 | High |
| A0A5F9ZH45 | PLEKHA6 | 0 | High |
| Q6P1J9 | CDC73 | 0 | High |
| O60231 | DHX16 | 0 | High |
| P62316 | SNRPD2 | 0 | High |
| Q8TAT6 | NPLOC4 | 0 | High |
| Q9NQW6 | ANLN | 0 | High |
| O43148 | RNMT | 0 | High |
| A0A0A0MTI1 | DHCR24 | 0 | High |
| Q86U44 | METTL3 | 0 | High |
| Q96EV2 | RBM33 | 0 | High |
| O60493 | SNX3 | 0 | High |
| P09327 | VIL1 | 0 | High |
| Q96QG7 | MTMR9 | 0 | High |
| A0A0U1RRM4 | PTBP1 | 0 | High |
| Q13177 | PAK2 | 0 | High |
| A0A6Q8PGS2 | GDAP1 | 0 | High |
| Q9H4Z3 | PCIF1 | 0 | High |
| P52594 | AGFG1 | 0 | High |
| O60271 | SPAG9 | 0 | High |
| Q13838 | DDX39B | 0 | High |
| P78386 | KRT85 | 0 | High |
| Q8NAV1 | PRPF38A | 0 | High |
| Q8TAF3 | WDR48 | 0 | High |
| A0A3B3ISG5 | IDE | 0 | High |
| P55268 | LAMB2 | 0 | High |
| Q05682 | CALD1 | 0 | High |
| Q14974 | KPNB1 | 0 | High |
| P02675 | FGB | 0 | High |
| Q9Y2W2 | WBP11 | 0 | High |
| O60547 | GMDS | 0 | High |
| O60506 | SYNCRIP | 0 | High |
| Q8IXI2 | RHOT1 | 0 | High |
| O15143 | ARPC1B | 0 | High |
| P13073 | COX4I1 | 0 | High |
| Q96FS4 | SIPA1 | 0 | High |
| Q5D862 | FLG2 | 0 | High |
| Q96AQ6 | PBXIP1 | 0 | High |
| O95757 | HSPA4L | 0 | High |
| P20930 | FLG | 0 | High |
| O75808 | CAPN15 | 0 | High |
| Q01469 | FABP5 | 0 | High |
| P27797 | CALR | 0 | High |
| P61964 | WDR5 | 0 | High |
| C9JIF9 | APEH | 0 | High |
| A0A499FI48 | PDIA4 | 0 | High |
| O43809 | NUDT21 | 0 | High |
| Q96B21 | TMEM45B | 0 | High |
| O14578 | CIT | 0 | High |
| Q13614 | MTMR2 | 0 | High |
| Q7Z434 | MAVS | 0 | High |
| Q10471 | GALNT2 | 0 | High |
| A0A0A0MQU4 | SCLY | 0 | High |
| Q14847 | LASP1 | 0 | High |
| P21281 | ATP6V1B2 | 0 | High |
| P99999 | CYCS | 0 | High |
| P49006 | MARCKSL1 | 0 | High |
| Q9NR19 | ACSS2 | 0 | High |
| Q9Y266 | NUDC | 0 | High |
| Q6KB66 | KRT80 | 0 | High |
| Q8TF74 | WIPF2 | 0 | High |
| P04792 | HSPB1 | 0 | High |
| P34810 | CD68 | 0 | High |
| Q12972 | PPP1R8 | 0 | High |
| Q9H1K1 | ISCU | 0 | High |
| Q8IYB5 | SMAP1 | 0 | High |
| H0YA82 | LARP7 | 0 | High |
| P05121 | SERPINE1 | 0 | High |
| P55084 | HADHB | 0 | High |
| K7ELG9 | LSM12 | 0 | High |
| P13984 | GTF2F2 | 0 | High |
| Q8TDB8 | SLC2A14 | 0 | High |
| P42785 | PRCP | 0 | High |
| P30048 | PRDX3 | 0 | High |
| Q9H1P3 | OSBPL2 | 0 | High |
| P16435 | POR | 0 | High |
| Q96F86 | EDC3 | 0 | High |
| A0A7I2YQK0 | GSK3B | 0 | High |
| A0A0U1RRH7 |  | 0 | High |
| P54709 | ATP1B3 | 0 | High |
| Q04837 | SSBP1 | 0 | High |
| O43776 | NARS1 | 0 | High |
| P23284 | PPIB | 0 | High |
| O15357 | INPPL1 | 0 | High |
| Q9BRQ8 | AIFM2 | 0 | High |
| Q9Y237 | PIN4 | 0 | High |
| P62805 | H4-16 | 0 | High |
| Q6UWP8 | SBSN | 0 | High |
| Q5RI15 | COX20 | 0 | High |
| Q8N5A5 | ZGPAT | 0 | High |
| Q8TAD8 | SNIP1 | 0 | High |
| P10909 | CLU | 0 | High |
| Q12834 | CDC20 | 0 | High |
| P31146 | CORO1A | 0 | High |
| P11387 | TOP1 | 0 | High |
| Q96ST2 | IWS1 | 0 | High |
| Q9H3K2 | GHITM | 0.001 | High |
| P11166 | SLC2A1 | 0.001 | High |
| A0A8I5KT88 | PDIA3 | 0.001 | High |
| Q9BY44 | EIF2A | 0.001 | High |
| Q9BUL5 | PHF23 | 0.001 | High |
| O60884 | DNAJA2 | 0.001 | High |
| Q99747 | NAPG | 0.001 | High |
| P07910 | HNRNPC | 0.001 | High |
| P04843 | RPN1 | 0.001 | High |
| Q15388 | TOMM20 | 0.001 | High |
| E7EX29 | YWHAZ | 0.001 | High |
| Q92620 | DHX38 | 0.001 | High |
| P14678 | SNRPB | 0.001 | High |
| P26572 | MGAT1 | 0.001 | High |
| Q8TD30 | GPT2 | 0.001 | High |
| P61803 | DAD1 | 0.001 | High |
| Q13485 | SMAD4 | 0.001 | High |
| Q7Z7F0 | KHDC4 | 0.001 | High |
| A0A1B0GW03 | PLEKHA6 | 0.001 | High |
| Q9BWS9 | CHID1 | 0.001 | High |
| Q9P2R6 | RERE | 0.001 | High |
| Q9H0C8 | ILKAP | 0.001 | High |
| O75131 | CPNE3 | 0.001 | High |
| A0A088AWN2 | WDR20 | 0.001 | High |
| F8WCF6 | ARPC4-TTLL3 | 0.001 | High |
| P62979 | RPS27A | 0.001 | High |
| Q8IVS2 | MCAT | 0.001 | High |
| Q15029 | EFTUD2 | 0.001 | High |
| P62136 | PPP1CA | 0.001 | High |
| Q96I25 | RBM17 | 0.001 | High |
| Q9NWH9 | SLTM | 0.001 | High |
| P62072 | TIMM10 | 0.001 | High |
| Q8NHV4 | NEDD1 | 0.001 | High |
| P52948 | NUP98 | 0.001 | High |
| Q8NBJ5 | COLGALT1 | 0.001 | High |
| Q92575 | UBXN4 | 0.001 | High |
| Q8TAP6 | CEP76 | 0.001 | High |
| U3KQK0 | H2BC15 | 0.001 | High |
| Q9Y2A7 | NCKAP1 | 0.001 | High |
| Q53EP0 | FNDC3B | 0.001 | High |
| Q09028 | RBBP4 | 0.001 | High |
| A0A8I5KW26 | CYB5R3 | 0.001 | High |
| Q9HDC9 | APMAP | 0.001 | High |
| B4E1Z4 |  | 0.001 | High |
| P08574 | CYC1 | 0.001 | High |
| Q9H0G5 | NSRP1 | 0.001 | High |
| Q9UKJ3 | GPATCH8 | 0.001 | High |
| Q93052 | LPP | 0.001 | High |
| A8MXP9 | MATR3 | 0.001 | High |
| Q8ND56 | LSM14A | 0.001 | High |
| H0YMW4 | ANXA2 | 0.001 | High |
| P01011 | SERPINA3 | 0.001 | High |
| P61981 | YWHAG | 0.001 | High |
| P09972 | ALDOC | 0.001 | High |
| A0A494C0J7 |  | 0.001 | High |
| Q99575 | POP1 | 0.001 | High |
| Q8IVM0 | CCDC50 | 0.001 | High |
| Q9ULW0 | TPX2 | 0.001 | High |
| O43615 | TIMM44 | 0.001 | High |
| Q9Y3A5 | SBDS | 0.001 | High |
| P31944 | CASP14 | 0.001 | High |
| Q6PI48 | DARS2 | 0.001 | High |
| O15371 | EIF3D | 0.001 | High |
| P02766 | TTR | 0.001 | High |
| P78504 | JAG1 | 0.001 | High |
| P15907 | ST6GAL1 | 0.001 | High |
| P60468 | SEC61B | 0.001 | High |
| P05154 | SERPINA5 | 0.001 | High |
| P53801 | PTTG1IP | 0.001 | High |
| P06746 | POLB | 0.001 | High |
| E9PB61 | ALYREF | 0.001 | High |
| Q9HA64 | FN3KRP | 0.001 | High |
| E9PKC0 | PLEKHA7 | 0.001 | High |
| Q92769 | HDAC2 | 0.001 | High |
| Q9H3F6 | KCTD10 | 0.001 | High |
| P62873 | GNB1 | 0.001 | High |
| P06576 | ATP5F1B | 0.001 | High |
| Q02809 | PLOD1 | 0.001 | High |
| Q92576 | PHF3 | 0.001 | High |
| P61956 | SUMO2 | 0.001 | High |
| Q14694 | USP10 | 0.001 | High |
| Q9UJW0 | DCTN4 | 0.001 | High |
| A0A0A0MRJ6 | PCMT1 | 0.001 | High |
| Q9Y295 | DRG1 | 0.001 | High |
| P60520 | GABARAPL2 | 0.001 | High |
| O75689 | ADAP1 | 0.001 | High |
| Q8IUW5 | RELL1 | 0.001 | High |
| E7ETB3 | DNPEP | 0.001 | High |
| A0A0U1RQM0 |  | 0.001 | High |
| Q9H307 | PNN | 0.001 | High |
| P49790 | NUP153 | 0.001 | High |
| P40926 | MDH2 | 0.001 | High |
| A0A7I2YQC0 | NPM1 | 0.001 | High |
| P00390 | GSR | 0.001 | High |
| Q9P2N5 | RBM27 | 0.001 | High |
| P43155 | CRAT | 0.001 | High |
| P31040 | SDHA | 0.001 | High |
| P18669 | PGAM1 | 0.001 | High |
| O15027 | SEC16A | 0.001 | High |
| F8W8Z9 | TOMM5 | 0.001 | High |
| P05109 | S100A8 | 0.001 | High |
| O94907 | DKK1 | 0.001 | High |
| Q9Y217 | MTMR6 | 0.001 | High |
| O15144 | ARPC2 | 0.001 | High |
| P52907 | CAPZA1 | 0.001 | High |
| P31947 | SFN | 0.001 | High |
| A6NEM2 | HCFC1 | 0.001 | High |
| P47929 | LGALS7B | 0.001 | High |
| P30419 | NMT1 | 0.001 | High |
| H3BMV3 | JPT2 | 0.001 | High |
| G3V2S9 | SLIRP | 0.001 | High |
| Q9NXR1 | NDE1 | 0.001 | High |
| P07737 | PFN1 | 0.001 | High |
| Q7Z7K0 | CMC1 | 0.001 | High |
| O76003 | GLRX3 | 0.001 | High |
| Q01081 | U2AF1 | 0.001 | High |
| Q96D05 | FAM241B | 0.001 | High |
| B1AK88 | CAPZB | 0.001 | High |
| O60443 | GSDME | 0.001 | High |
| A0A7P0Z4P5 | SLC3A2 | 0.001 | High |
| P02792 | FTL | 0.001 | High |
| O95486 | SEC24A | 0.001 | High |
| A0A0G2JPD3 | HLA-A | 0.001 | High |
| P48436 | SOX9 | 0.001 | High |
| P20618 | PSMB1 | 0.001 | High |
| Q14241 | ELOA | 0.001 | High |
| Q01518 | CAP1 | 0.001 | High |
| J3KR35 | CCDC12 | 0.001 | High |
| Q9HB66 | MKKS | 0.001 | High |
| H3BQK9 | MACF1 | 0.001 | High |
| A0A0U1RRM6 | ENAH | 0.001 | High |
| Q92890 | UFD1 | 0.001 | High |
| Q08554 | DSC1 | 0.001 | High |
| Q86YM7 | HOMER1 | 0.001 | High |
| P22528 | SPRR1B | 0.001 | High |
| P55010 | EIF5 | 0.001 | High |
| O94876 | TMCC1 | 0.001 | High |
| P38159 | RBMX | 0.001 | High |
| Q8WWI1 | LMO7 | 0.001 | High |
| Q13547 | HDAC1 | 0.001 | High |
| A0A0A0MQU6 | SEMA6A | 0.001 | High |
| Q9BWU0 | SLC4A1AP | 0.001 | High |
| Q9UBU9 | NXF1 | 0.001 | High |
| Q9C0J8 | WDR33 | 0.001 | High |
| P00734 | F2 | 0.001 | High |
| O00469 | PLOD2 | 0.001 | High |
| P14927 | UQCRB | 0.001 | High |
| Q3YEC7 | RABL6 | 0.001 | High |
| Q9NUK0 | MBNL3 | 0.001 | High |
| Q15125 | EBP | 0.001 | High |
| P48426 | PIP4K2A | 0.001 | High |
| Q9Y5K6 | CD2AP | 0.001 | High |
| Q2Q1W2 | TRIM71 | 0.001 | High |
| A0A8I5KW48 | PTPN11 | 0.001 | High |
| P62304 | SNRPE | 0.001 | High |
| P00505 | GOT2 | 0.001 | High |
| O15145 | ARPC3 | 0.001 | High |
| Q6UW68 | TMEM205 | 0.001 | High |
| P60763 | RAC3 | 0.001 | High |
| Q9BQ61 | TRIR | 0.001 | High |
| Q14554 | PDIA5 | 0.001 | High |
| P04156 | PRNP | 0.001 | High |
| Q96S94 | CCNL2 | 0.001 | High |
| P02751 | FN1 | 0.001 | High |
| P34896 | SHMT1 | 0.001 | High |
| Q92804 | TAF15 | 0.001 | High |
| P26639 | TARS1 | 0.001 | High |
| Q6P1L8 | MRPL14 | 0.001 | High |
| P09622 | DLD | 0.001 | High |
| Q01844 | EWSR1 | 0.001 | High |
| A0A590UJD9 | DLG1 | 0.001 | High |
| P61769 | B2M | 0.001 | High |
| P17980 | PSMC3 | 0.001 | High |
| U3KQB3 | CSNK1G1 | 0.001 | High |
| P10599 | TXN | 0.001 | High |
| A0A087X0H9 | RBM26 | 0.001 | High |
| P27540 | ARNT | 0.001 | High |
| P30408 | TM4SF1 | 0.001 | High |
| P60059 | SEC61G | 0.001 | High |
| Q05048 | CSTF1 | 0.001 | High |
| A0A0A0MQV6 | FGF2 | 0.001 | High |
| Q86UY8 | NT5DC3 | 0.001 | High |
| Q9NQ29 | LUC7L | 0.001 | High |
| A0A7P0T8D1 | AGT | 0.001 | High |
| P02786 | TFRC | 0.001 | High |
| Q9UMS4 | PRPF19 | 0.001 | High |
| Q15043 | SLC39A14 | 0.001 | High |
| A0A804HLA8 | GNG5P2 | 0.001 | High |
| A0A2R8YDB8 | SAP130 | 0.001 | High |
| Q86WR7 | PROSER2 | 0.001 | High |
| P39060 | COL18A1 | 0.004 | High |
| G3V5Z7 | PSMA6 | 0.004 | High |
| P49720 | PSMB3 | 0.004 | High |
| J3KPP4 | LUC7L3 | 0.004 | High |
| O15127 | SCAMP2 | 0.004 | High |
| Q9NPJ3 | ACOT13 | 0.004 | High |
| P06396 | GSN | 0.004 | High |
| P62195 | PSMC5 | 0.004 | High |
| P02647 | APOA1 | 0.004 | High |
| Q96HR9 | REEP6 | 0.004 | High |
| P16144 | ITGB4 | 0.004 | High |
| Q9ULF5 | SLC39A10 | 0.003 | High |
| O60942 | RNGTT | 0.003 | High |
| P52657 | GTF2A2 | 0.003 | High |
| E9PDI4 | LAD1 | 0.003 | High |
| P25398 | RPS12 | 0.003 | High |
| B0YIW6 | ARCN1 | 0.003 | High |
| Q9H0E2 | TOLLIP | 0.003 | High |
| O95197 | RTN3 | 0.003 | High |
| Q9ULR3 | PPM1H | 0.003 | High |
| P09661 | SNRPA1 | 0.003 | High |
| P25685 | DNAJB1 | 0.003 | High |
| P06703 | S100A6 | 0.003 | High |
| Q9BZL1 | UBL5 | 0.003 | High |
| Q13268 | DHRS2 | 0.003 | High |
| P27694 | RPA1 | 0.003 | High |
| Q96FQ6 | S100A16 | 0.003 | High |
| P28332 | ADH6 | 0.003 | High |
| Q9H098 | FAM107B | 0.003 | High |
| Q8TCD1 | C18orf32 | 0.003 | High |
| Q8IYD1 | GSPT2 | 0.003 | High |
| Q86UU0 | BCL9L | 0.003 | High |
| P35269 | GTF2F1 | 0.003 | High |
| Q2T9J0 | TYSND1 | 0.003 | High |
| Q99436 | PSMB7 | 0.003 | High |
| Q06203 | PPAT | 0.003 | High |
| P11279 | LAMP1 | 0.003 | High |
| A0A087X1Z3 | PSME2 | 0.003 | High |
| Q9Y2L1 | DIS3 | 0.003 | High |
| B9ZVT1 | RBM12B | 0.003 | High |
| Q07666 | KHDRBS1 | 0.003 | High |
| I1E4Y6 | GIGYF2 | 0.003 | High |
| Q14103 | HNRNPD | 0.003 | High |
| Q9UJM3 | ERRFI1 | 0.003 | High |
| E9PGN7 | SERPING1 | 0.003 | High |
| B8ZZS0 | BET1L | 0.003 | High |
| Q8N9N8 | EIF1AD | 0.003 | High |
| Q13627 | DYRK1A | 0.003 | High |
| Q14258 | TRIM25 | 0.003 | High |
| P30405 | PPIF | 0.003 | High |
| Q8TBQ9 | TMEM167A | 0.003 | High |
| P05089 | ARG1 | 0.003 | High |
| Q75N03 | CBLL1 | 0.003 | High |
| P12270 | TPR | 0.003 | High |
| Q92520 | FAM3C | 0.003 | High |
| Q9Y6A9 | SPCS1 | 0.004 | High |
| P27105 | STOM | 0.005 | High |
| Q5SRQ6 | CSNK2B | 0.005 | High |
| O75348 | ATP6V1G1 | 0.005 | High |
| P54259 | ATN1 | 0.005 | High |
| P52209 | PGD | 0.005 | High |
| Q8N4T8 | CBR4 | 0.005 | High |
| P61978 | HNRNPK | 0.005 | High |
| P13473 | LAMP2 | 0.005 | High |
| P21397 | MAOA | 0.005 | High |
| H3BNX8 | COX5A | 0.005 | High |
| O43920 | NDUFS5 | 0.005 | High |
| F5H039 | GPHN | 0.005 | High |
| A0A7P0TB81 | IFITM2 | 0.005 | High |
| Q9HD15 | SRA1 | 0.005 | High |
| Q9NPA8 | ENY2 | 0.007 | High |
| Q15019 | SEPTIN2 | 0.007 | High |
| P42167 | TMPO | 0.007 | High |
| P26196 | DDX6 | 0.008 | High |
| P55957 | BID | 0.008 | High |
| O95562 | SFT2D2 | 0.008 | High |
| Q9UFN0 | NIPSNAP3A | 0.008 | High |
| P18858 | LIG1 | 0.008 | High |
| P11172 | UMPS | 0.008 | High |
| P38117 | ETFB | 0.008 | High |
| X6R4W8 | ZNF207 | 0.008 | High |
| Q9Y3F4 | STRAP | 0.008 | High |
| A0A1B0GVP4 | LCORL | 0.008 | High |
| A0A087WX23 | PEG10 | 0.009 | High |
| Q9HBH1 | PDF | 0.009 | High |
| Q9P206 | KIAA1522 | 0.009 | High |
| O76021 | RSL1D1 | 0.009 | High |
| P41091 | EIF2S3 | 0.009 | High |
| O94762 | RECQL5 | 0.009 | High |
| P61604 | HSPE1 | 0.009 | High |
| O95297 | MPZL1 | 0.009 | High |
| P61962 | DCAF7 | 0.009 | High |
| A1L0T0 | ILVBL | 0.009 | High |
| Q9BU61 | NDUFAF3 | 0.009 | High |
| P29350 | PTPN6 | 0.01 | High |
| A0A7I2V5M7 | G3BP1 | 0.01 | High |
| Q8IYB8 | SUPV3L1 | 0.01 | High |
| A0A2R8Y5Q8 | TBCE | 0.01 | High |
| Q9C037 | TRIM4 | 0.01 | Medium |
| O14874 | BCKDK | 0.01 | Medium |
| Q9BRL6 | SRSF8 | 0.01 | Medium |
| O15213 | WDR46 | 0.014 | Medium |
| F5H5D3 | TUBA1C | 0.014 | Medium |
| Q8N5I2 | ARRDC1 | 0.014 | Medium |
| P49908 | SELENOP | 0.014 | Medium |
| P35568 | IRS1 | 0.014 | Medium |
| A0A7P0T8E1 | OAS1 | 0.014 | Medium |
| Q5EBL8 | PDZD11 | 0.014 | Medium |
| Q9GZZ1 | NAA50 | 0.014 | Medium |
| Q9H7Z7 | PTGES2 | 0.014 | Medium |
| Q6YN16 | HSDL2 | 0.014 | Medium |
| Q9UGJ1 | TUBGCP4 | 0.014 | Medium |
| P51148 | RAB5C | 0.014 | Medium |
| Q8WUR7 | C15orf40 | 0.014 | Medium |
| Q8IYS2 | KIAA2013 | 0.015 | Medium |
| P09651 | HNRNPA1 | 0.015 | Medium |
| Q8NDI1 | EHBP1 | 0.015 | Medium |
| A0A7I2PJZ2 | MAPT | 0.015 | Medium |
| Q9GZL7 | WDR12 | 0.015 | Medium |
| Q9Y5U9 | IER3IP1 | 0.015 | Medium |
| P31151 | S100A7 | 0.015 | Medium |
| Q92466 | DDB2 | 0.015 | Medium |
| P48449 | LSS | 0.015 | Medium |
| A0A024R6I7 | SERPINA1 | 0.015 | Medium |
| A0A075B6Z2 | TRAJ56 | 0.015 | Medium |
| Q66LE6 | PPP2R2D | 0.015 | Medium |
| Q96A05 | ATP6V1E2 | 0.015 | Medium |
| P61313 | RPL15 | 0.015 | Medium |
| P02749 | APOH | 0.015 | Medium |
| B4DUT8 | CNN2 | 0.015 | Medium |
| Q9UGU0 | TCF20 | 0.015 | Medium |
| Q9Y4B5 | MTCL1 | 0.015 | Medium |
| P01040 | CSTA | 0.015 | Medium |
| O95405 | ZFYVE9 | 0.015 | Medium |
| Q96HE9 | PRR11 | 0.015 | Medium |
| Q9NYZ3 | GTSE1 | 0.015 | Medium |
| O15198 | SMAD9 | 0.015 | Medium |
| C9JF17 | APOD | 0.015 | Medium |
| A6ND36 | FAM83G | 0.016 | Medium |
| O76061 | STC2 | 0.016 | Medium |
| P62266 | RPS23 | 0.016 | Medium |
| P02788 | LTF | 0.016 | Medium |
| Q5SW79 | CEP170 | 0.016 | Medium |
| A0A7I2V3E8 | CTSH | 0.016 | Medium |
| Q8WU90 | ZC3H15 | 0.017 | Medium |
| P28072 | PSMB6 | 0.017 | Medium |
| P37837 | TALDO1 | 0.017 | Medium |
| P01031 | C5 | 0.017 | Medium |
| Q9UPN9 | TRIM33 | 0.017 | Medium |
| Q7RTV0 | PHF5A | 0.017 | Medium |
| P49411 | TUFM | 0.016 | Medium |
| Q8NDH2 | CCDC168 | 0.016 | Medium |
| Q9BWH2 | FUNDC2 | 0.016 | Medium |
| Q69YU5 | BRAWNIN | 0.016 | Medium |
| Q9NW64 | RBM22 | 0.016 | Medium |
| P09669 | COX6C | 0.016 | Medium |
| Q9BRR6 | ADPGK | 0.016 | Medium |
| Q6VY07 | PACS1 | 0.016 | Medium |
| Q9HCN8 | SDF2L1 | 0.016 | Medium |
| Q04637 | EIF4G1 | 0.016 | Medium |
| A0A2R8Y3M9 |  | 0.017 | Medium |
| Q15942 | ZYX | 0.017 | Medium |
| P04196 | HRG | 0.017 | Medium |
| Q8TDW7 | FAT3 | 0.017 | Medium |
| A0A804HJ20 | AFDN | 0.017 | Medium |
| Q5T7N2 | L1TD1 | 0.017 | Medium |
| A0A0A0MT33 | SCAF8 | 0.017 | Medium |
| Q53S33 | BOLA3 | 0.017 | Medium |
| Q9BQ75 | CMSS1 | 0.017 | Medium |
| P01042 | KNG1 | 0.017 | Medium |
| A0A7N4I394 | PRPF40A | 0.017 | Medium |
| A0A8I5KNT2 | ATP1B1 | 0.018 | Medium |
| A0A1B0GW23 | ABHD14A-ACY1 | 0.018 | Medium |
| Q15758 | SLC1A5 | 0.018 | Medium |
| Q9P2X0 | DPM3 | 0.018 | Medium |
| H3BPG6 | ZNF598 | 0.018 | Medium |
| Q8N543 | OGFOD1 | 0.018 | Medium |
| Q9Y5J6 | TIMM10B | 0.018 | Medium |
| P27708 | CAD | 0.018 | Medium |
| Q5SYE7 | NHSL1 | 0.018 | Medium |
| Q9NPH2 | ISYNA1 | 0.018 | Medium |
| O75947 | ATP5PD | 0.018 | Medium |
| Q8IWE2 | FAM114A1 | 0.018 | Medium |
| Q8N1G4 | LRRC47 | 0.018 | Medium |
| P61457 | PCBD1 | 0.02 | Medium |
| P37108 | SRP14 | 0.02 | Medium |
| Q9Y697 | NFS1 | 0.02 | Medium |
| A0A087X2I1 | PSMC6 | 0.02 | Medium |
| Q9NZB2 | FAM120A | 0.02 | Medium |
| P08559 | PDHA1 | 0.02 | Medium |
| P12074 | COX6A1 | 0.02 | Medium |
| Q9UKM9 | RALY | 0.022 | Medium |
| Q96P63 | SERPINB12 | 0.023 | Medium |
| A0A0D9SFM0 | SHPRH | 0.023 | Medium |
| Q9UBV8 | PEF1 | 0.023 | Medium |
| A0A1B0GU03 |  | 0.023 | Medium |
| Q9Y624 | F11R | 0.023 | Medium |
| O00233 | PSMD9 | 0.023 | Medium |
| A2A274 | ACO2 | 0.023 | Medium |
| G0XQ39 | STIM1 | 0.022 | Medium |
| Q2VYF4 | LETM2 | 0.022 | Medium |
| C9JPK5 | ITGB1 | 0.022 | Medium |
| Q6GQQ9 | OTUD7B | 0.022 | Medium |
| P11233 | RALA | 0.022 | Medium |
| A0A804HJC8 | STAMBP | 0.022 | Medium |
| Q13033 | STRN3 | 0.023 | Medium |
| Q4G0I0 | CCSMST1 | 0.023 | Medium |
| Q8NFC6 | BOD1L1 | 0.023 | Medium |
| Q8N2G8 | GHDC | 0.023 | Medium |
| A0A8I5KQV3 | KIAA1109 | 0.023 | Medium |
| Q8NF91 | SYNE1 | 0.023 | Medium |
| Q99873 | PRMT1 | 0.023 | Medium |
| Q9BYE4 | SPRR2G | 0.024 | Medium |
| A0A804HKA2 | PPFIBP2 | 0.024 | Medium |
| Q6ZVX7 | NCCRP1 | 0.024 | Medium |
| Q86UW9 | DTX2 | 0.025 | Medium |
| K7ELL7 | PRKCSH | 0.025 | Medium |
| Q9NV31 | IMP3 | 0.025 | Medium |
| Q99988 | GDF15 | 0.025 | Medium |
| P10632 | CYP2C8 | 0.025 | Medium |
| Q9P021 | CRIPT | 0.025 | Medium |
| Q86W56 | PARG | 0.025 | Medium |
| O60828 | PQBP1 | 0.025 | Medium |
| Q8WW27 | APOBEC4 | 0.025 | Medium |
| Q12874 | SF3A3 | 0.025 | Medium |
| Q99460 | PSMD1 | 0.025 | Medium |
| Q08257 | CRYZ | 0.025 | Medium |
| Q16775 | HAGH | 0.025 | Medium |
| Q13835 | PKP1 | 0.025 | Medium |
| Q9H479 | FN3K | 0.03 | Medium |
| Q9Y6X8 | ZHX2 | 0.03 | Medium |
| Q16543 | CDC37 | 0.03 | Medium |
| Q9Y4P3 | TBL2 | 0.03 | Medium |
| P42765 | ACAA2 | 0.03 | Medium |
| A0A8C8L3F5 | ACSL5 | 0.03 | Medium |
| P04083 | ANXA1 | 0.03 | Medium |
| P54278 | PMS2 | 0.031 | Medium |
| A0A5F9ZGX9 | BBS9 | 0.031 | Medium |
| A0A5H1ZRQ2 | DDX17 | 0.032 | Medium |
| A0A0J9YWL0 | CRYBG1 | 0.032 | Medium |
| A0A8I5KZ24 | ATE1 | 0.032 | Medium |
| Q8TEW0 | PARD3 | 0.031 | Medium |
| P29508 | SERPINB3 | 0.031 | Medium |
| Q86Y26 | NUTM1 | 0.031 | Medium |
| X6R8A1 | CTSA | 0.032 | Medium |
| Q99961 | SH3GL1 | 0.032 | Medium |
| P62495 | ETF1 | 0.032 | Medium |
| P51956 | NEK3 | 0.038 | Medium |
| O14976 | GAK | 0.038 | Medium |
| Q68CQ4 | UTP25 | 0.039 | Medium |
| P56385 | ATP5ME | 0.039 | Medium |
| P35613 | BSG | 0.039 | Medium |
| Q6P1N0 | CC2D1A | 0.04 | Medium |
| Q9NTZ6 | RBM12 | 0.04 | Medium |
| H3BTB6 | CMC2 | 0.04 | Medium |
| A0A7I2V4I5 | ZC3H13 | 0.04 | Medium |
| Q86XJ1 | GAS2L3 | 0.04 | Medium |

**Table S3. Primer sequences used in the study are shown as follows**

| **Human gene** | **Forward primer** | **Reverse primer** |
| --- | --- | --- |
| PPARα | CTGTCGGGATGTCACACAAC | CGGGCTTTGACCTTGTTCAT |
| CPT1A | AGTTCTCTTGCCCTGAGACG | TTCCAGCCCAGCACATGAAC |
| ACOX1 | CCGCCACCTTCAA TCCAGAG | CAAGTTCTCGATTTCTCGACGG |
| MCAD | GGCCGTGACCCGTGTATTAT | CTGCAGCATCGCCCGAA |
| LCAD | ATTGTCTGGGAGGAGCAAGC | CTCCAGGCTCTGTCATTGCT |
| FASN | CTGCCAAGAGAGGAAGGAGT | ACATTTGGTGCAAGGGTCAC |
| ATGL | TCTCAGGCGAGAGTGACATC | CCCTGTTTGCACATCTCTCG |
| ACLY | GACTTCGGCAGAGGTAGAGC | TCAGGAGTGACCCGAGCATA |
| β-actin | ATTGGCAACGAGCGGTTCC | AGCACTGTGTTGGCATAGAGG |
| Asprosin | CACAAGCGGACAGGAGCC | CTGCTCCCAGTCGTGGC |
| SCD1 | AAACAGTGTGTTCGTTGCCA | GGAGTGGTGGTAGTTGTGGA |
| TNFα | CACAGTGAAGTGCTGGCAAC | AGGAAGGCCTAAGGTCCACT |
| Ucp2 | CTCTGGCCTTCACAACATCCT | AGGCTCAGGCAAATGGTATCC |
| OLFR734 | CTCAGACTCGGGAAGTGCAG | AAGCATCTTAGGCGCTGTGA |
| FABP5 | TTCAGCAGCTGGAAGGAAGA | CGCAAAGCTATTCCCACTCC |
| **Mouse gene** | **Forward primer** | **Reverse primer** |
| PPARα | GCATGTGAAGGCTGTAAGGG | TTGTGTGACATCCCCGACAGA |
| CPT1A | GGACTCCGCTCGCTCATT | GAGATCGATGCCATCAGGGG |
| ACOX1 | GCCATTCGATACAGTHCTGTGAG | CCGAGAAAGTGGAAGGCATAGG |
| MCAD | AACTAAACATGGGCCAGCGA | GAAACCTGCTCCTTCACCGA |
| LCAD | GTCCGATTGCCAGCTAATGC | CACAGGCAGAAATCGCCAAC |
| FASN | GGAGGCGGGTTCGTGAAACTG | AACGGGCTGAATTTCTGATGGTCTC |
| ATGL | TCTCAGGCGAGAGTGACATC | CCCTGTTTGCACATCTCTCG |
| ACLY | TAGAGCTCAGGCTAGGGAACG | GTGTGGCTCTCTGTCCGTAA |
| β-actin | ATGCCACAGGATTCCATACCCAAGA | CTCTAGACTTCGAGCAGGAGATGG |
| Asprosin | CTGAGATGGAAGCCAACGTGAGCCT | TGTGGTTCATCAGAGTTGTGAGGGC |
| SCD1 | CCTTCGACTACTCTGCCAGT | GAAGCCCAAAGCTCAGCTAC |
| TNFα | ACTGAACTTCGGGGTGATCG | TGGTGGTTTGTGAGTGTGAGG |
| IL-1β | TGCCACCTTTTGACAGTGATG | TGATGTGCTGCGGAGATT |
| COL3A1 | GCGAGCGGCTGAGTTTTATG | GCAGCTCAGAGTAGCACCAT |
| CCL2 | TGCCCTAAGGTCTTCAGCAC | AAGGCATCCACGTCCGAGTC |
| COL1A1 | TTCTCCTGGCAAAGACGGAC | CGGCCACCATCTTGAGACTT |
| UCP2 | CTCCAGGCTCTGTCATTGCT | GGAGTTCTGGAGGCTGCTTT |
| OLFR734 | CTCAGGTTGTCCGGATTGCT | GTGCTCTCACCTGAGCTTGA |
| FABP5 | ATGGCCAAGCCAGACTGTAT | TCTTCACTGTGCTCTCGGTT |
